# Synergistic Configuration of Binary Rhodium Single Atoms in Carbon Nanofibers for High‐Performance Alkaline Water Electrolyzer

**DOI:** 10.1002/advs.202413176

**Published:** 2024-11-24

**Authors:** Natarajan Logeshwaran, Gyuchan Kim, Pandiarajan Thangavel, Sun Seo Jeon, Kaliannan Thiyagarajan, Kampara Roopa Kishore, Hyunjoo Lee, Inseok Seo, Hongseok Yun, Sungho Lee, Byung‐Hyun Kim, Young Jun Lee

**Affiliations:** ^1^ Carbon Composite Materials Research Center Korea Institute of Science and Technology (KIST) 92 Chudong‐ro, Bongdong‐eup Wanju‐gun Jeonbuk 55324 Republic of Korea; ^2^ Department of Applied Chemistry Center for Bionano Intelligence Education and Research Hanyang University ERICA 55 Hanyangdaehak‐ro, Sangnok‐gu Ansan‐si Gyeonggi‐do 15588 Republic of Korea; ^3^ Department of Chemistry Ulsan National Institute of Science and Technology (UNIST) UNIST‐gil, Eonyang‐eup, Ulju‐gun Ulsan 44919 Republic of Korea; ^4^ Department of Chemical and Biomolecular Engineering Korea Advanced Institute of Science and Technology (KAIST) Daejeon 34141 Republic of Korea; ^5^ Department of Mechanical Engineering Ulsan National Institute of Science and Technology (UNIST) UNIST‐gil, Eonyang‐eup, Ulju‐gun Ulsan 44919 Republic of Korea; ^6^ School of Advanced Materials Engineering Jeonbuk National University Baekje‐daero 567 Jeonju 54896 Republic of Korea; ^7^ Department of Chemistry Hanyang University 222, Wangsimni‐ro, Seongdong‐gu Seoul Republic of Korea

**Keywords:** anion exchange membrane water electrolyzer, carbon nanofiber, hydrogen evolution reaction, interface engineering, single atom dispersion

## Abstract

Electrochemical alkaline water electrolysis offers significant economic advantages; however, these benefits are hindered by the high kinetic energy barrier of the water dissociation step and the sluggish kinetics of the hydrogen evolution reaction (HER) in alkaline media. Herein, the ensemble effect of binary types of Rh single atoms (Rh‐N_x_ and Rh‐O_x_) on TiO_2_‐embedded carbon nanofiber (Rh‐TiO_2_/CNF) is reported, which serves as potent active sites for high‐performance HER in anion exchange membrane water electrolyzer (AEMWE). Density functional theory (DFT) analyses support the experimental observations, highlighting the critical role of binary types of Rh single atoms facilitated by the TiO_2_ sites. The Rh‐TiO_2_/CNF demonstrates an impressive areal current density of 1 A cm^−2^, maintaining extended durability for up to 225 h in a single‐cell setup. Furthermore, a 2‐cell AEMWE stack utilizing Rh‐TiO_2_/CNF is tested under industrial‐scale conditions. This research makes a significant contribution to the commercialization of next‐generation high‐performance and durable AEMWE stacks for clean hydrogen production.

## Graphical abstract

The Rh‐TiO_2_/CNF catalyst is presented, featuring binary Rh SAs (Rh‐N_x_ and Rh‐O_x_) as potent active sites for enhanced HER in AEMWE. The catalyst achieves an impressive areal current density of 1 A cm⁻^2^, demonstrating extended durability in a single‐cell setup. Its performance is further validated in a 2‐cell AEMWE stack under industrial‐scale conditions.

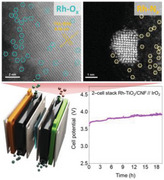

## 1 Introduction

Since the Industrial Revolution, the demand for clean, reliable, and affordable energy sources has intensified due to the depletion of fossil fuels. Recently, electrochemical water electrolysis has been recognized as a promising carbon‐free method for hydrogen (H_2_) production.^[^ ^1^, ^2^, ^3^, ^4^, ^5^, ^6^, ^7^ ^]^ This process can be conducted in either acidic or alkaline media. However, because most catalysts tend to corrode in acidic environments, anion exchange membrane water electrolyzers (AEMWEs) operating in alkaline media present a viable solution for industrial‐scale H_2_ production with a variety of materials.^[^ ^8^ ^]^ Despite extensive research efforts over the past several decades to identify cost‐effective alternative catalysts, platinum (Pt) remains the most efficient material for the hydrogen evolution reaction (HER) in AEMWEs. However, commercial Pt/C catalysts face significant challenges, including high cost, low mass activity for HER in alkaline media, and insufficient stability.^[^ ^9^, ^10^ ^]^ These limitations hinder the widespread adoption of Pt catalysts in AEMWEs for large‐scale applications. As a result, intensive research has focused on modifying noble catalysts by incorporating transition metals such as cobalt (Co), nickel (Ni), and iron (Fe).^[^ ^11^, ^12^, ^13^, ^14^, ^15^ ^]^ Additionally, various modifications to the support materials are being explored, including interface chemistry, defect engineering, doping strategies, and electronic structural modulation.^[^ ^16^, ^17^ ^]^

Recent trends indicate a growing interest in electrocatalytic regulation at the atomic scale, particularly in the configurations of single atom (SA) phases.^[^ ^18^, ^19^, ^20^, ^21^ ^]^ This innovative approach aims to enhance utilization in a cost‐effective manner while maintaining catalytic efficiency, making it an ideal platform for energy conversion applications. However, SA configuration suffers from a low density of active sites, which hinders their ability to efficiently facilitate the transfer of multiple electrons and protons, thereby limiting their overall catalytic performance.^[^ ^22^, ^23^ ^]^ Recent studies have revealed that binary or dual SA provides greater synergy than SA alone.^[^ ^24^, ^25^, ^26^ ^]^ In alkaline HER, the synergistic coexistence of binary or dual SA facilitates the Tafel‐Volmer mechanism, enhancing the adsorption of hydrogen atoms and water molecules, which leads to significantly improved catalytic performance.^[^ ^24^, ^25^, ^26^ ^]^ However, to date, most research has focused on binary SAs utilizing two or more metals, while studies on controlling SA configurations with only a single metal have been rarely conducted.

Rhodium (Rh) has emerged as a promising alternative to Pt in electrocatalysis for the HER.^[^ ^27^, ^28^, ^29^ ^]^ Although Rh exhibits performance comparable to that of Pt, its more favorable Gibbs free energy (ΔG) value for the adsorption of atomic hydrogen may impede the HER. To address this, Rh catalysts are often paired with supports that exhibit moderate ΔG values to enhance both stability and intrinsic HER properties. Among these supports, 3d transition metal oxides, particularly titanium oxides (TiO_2_), have garnered significant interest.^[^ ^30^, ^31^, ^32^, ^33^, ^34^ ^]^ These materials are highly appealing for research due to their tunable electronic structures, advantageous semiconductor properties, non‐toxic nature, chemical corrosion resistance, and durability.^[^ ^33^, ^34^ ^]^ These characteristics are crucial for facilitating stronger charge transfer (CT), providing larger active sites for support, and controlling the aggregation of high‐energy metal particles. Moreover, integrating high‐conductivity supports like carbon nanofibers (CNF) derived from polymers or pitch can produce mechanically flexible conductive materials, further enhancing electrochemical capabilities. The high conductivity of CNF itself serves as an efficient pathway for electron transfer during electrochemical reactions, facilitating rapid and stable electron mobility across the structure.^[^ ^35^, ^36^, ^37^ ^]^

In this study, we employ an electrospinning technique to achieve the spontaneous integration of dual Rh SAs (Rh‐N_x_ and Rh‐O_x_) within a TiO_2_‐embedded CNF composite (Rh‐TiO_2_/CNF). This material exhibits substantial potential as an innovative and efficient electrocatalyst for the HER. The unique configuration of Rh‐N_x_ and Rh‐O_x_ induces a synergistic enhancement in the Volmer‐Tafel kinetics, thereby significantly improving HER performance. In a single‐cell setup, the Rh‐TiO_2_/CNF reaches an industrial‐scale current density of 1 A cm^−2^, demonstrating extended durability of 225 h. Furthermore, theoretical analysis reveals that the combined hydrogen and water adsorption capabilities of the dual SA phases result in considerably lower energy barriers for HER, particularly when Rh‐N_x_ and Rh‐O_x_ coexist. Moreover, we test a 2‐cell AEMWE stack using Rh‐TiO_2_/CNF, which demonstrates an areal current density of 1.5 A cm^−^^2^ and stable performance over 19.5 h. These findings underscore the promise of Rh‐TiO_2_/CNF as a highly effective electrocatalyst for HER in alkaline media.

## 2 Results and Discussion

### 2.1 Synthesis and Characterization of Rh‐TiO_2_/CNF

**Figure 1:**
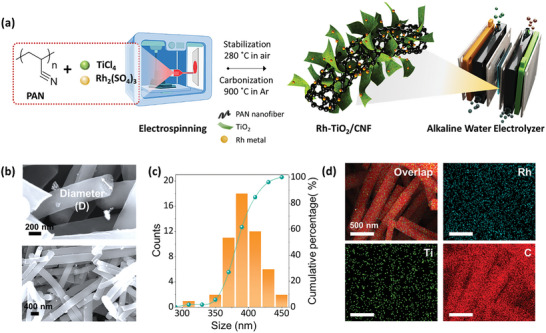
a) Schematic illustration of Rh‐TiO_2_/CNF fabrication and its application in AEMWE. b) SEM images of Rh‐TiO_2_/CNF, c) average nanofiber diameter histogram, and d) EDS elemental mapping images of Rh, Ti, O, and C in Rh‐TiO_2_/CNF.

**Figure** **1**a illustrates the comprehensive synthetic procedure for the Rh‐TiO_2_/CNF, which is designed as a HER catalyst. As detailed in the **Experimental section**, polyacrylonitrile (PAN) nanofibers embedded with Rh and Ti precursors were prepared using the electrospinning method. Subsequent carbonization of these stabilized PAN nanofibers resulted in the formation of Rh‐TiO_2_/CNF, where binary types of SA and nanoparticles coexisted within the CNF. This approach of directly incorporating metal precursors into the electrospinning process offers notable advantages, including more uniform nanofiber formation and a one‐pot synthesis. Figure 1b shows the scanning electron microscopy (SEM) images of the Rh‐TiO_2_/CNF, with an average diameter of 380 nm (Figure 1c). The average diameter was calculated by analyzing more than 100 measurements from SEM images. Energy‐dispersive X‐ray spectroscopy (EDS) mapping and SEM line scan analysis confirmed that Rh and Ti were evenly distributed throughout the CNF matrix (Figure 1d; Figure S1, Supporting Information). To elucidate the roles of Rh and Ti, TiO_2_/CNF and CNF were selected as comparison groups. The morphologies of TiO_2_/CNF and CNF were also analyzed using SEM, EDS mappings, diameter analysis, and line scan spectra, as shown in Figures S2–S4 (Supporting Information).

**Figure 2:**
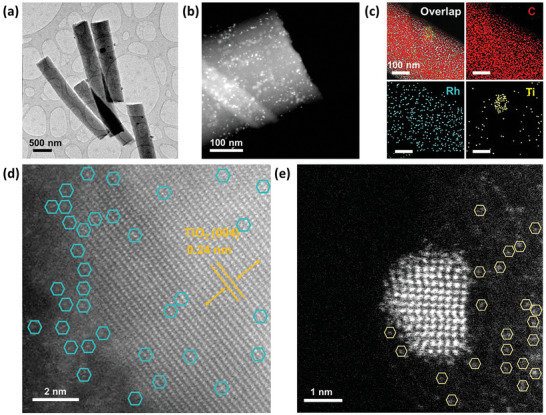
Morphological characterization: a) TEM, b) HAADF‐STEM, c) EDS mapping images, d, e) atomic scale STEM images of Rh‐TiO_2_/CNF: d) Rh‐O_x_ and e) Rh‐N_x_.

Compositional characterization was further conducted using transmission electron microscopy (TEM) and high‐angle annular dark‐field scanning transmission electron microscopy (HAADF‐STEM) images. The TEM and HAADF‐STEM images show that numerous SAs and nanoparticles coexist on the surface of the CNF (**Figure** **2**a,b). EDS mapping in Figure 2c further demonstrates a homogeneous distribution of Rh across the CNF, with Ti exhibiting slight aggregation into larger nanoparticles. The high‐resolution (HR)‐STEM image in Figure 2d confirms the alignment of Rh SA along TiO_2_. As discussed in the subsequent characterization section, Rh interacts exclusively with oxygen in TiO_2_, forming SAs such as Rh‐O_x_. The HR‐STEM image in Figure 2e highlights the dispersion of isolated Rh SAs alongside Rh nanoparticles on the CNF matrix, where the interaction between N on CNF and Rh results in the formation of SAs, such as Rh‐N_x_. This confirms the simultaneous presence of binary SA phases (i.e., Rh‐N_x_ and Rh‐O_x_). A minimal number of nanoparticles is inevitably formed; however, given the low loading, their overall contribution remains negligible relative to the generated specific surface area. This strategic distribution of Rh phases within the TiO_2_/CNF heterostructure is poised to significantly enhance the synergy in H^*^ adsorption and OH^−^ desorption kinetics, thereby efficiently accelerating water dissociation reactions, as further discussed in the DFT calculation section. Additional TEM images of TiO_2_/CNF are available in Figure S5 (Supporting Information). Elemental compositional weight percentages were accurately measured using inductively coupled plasma‐optical emission spectrometry (ICP‐OES), revealing significant Rh and Ti loading weights of 1.8% and 2.4% respectively, consistent with STEM EDS results shown in Table S1 (Supporting Information). Elemental analysis (EA) revealed nitrogen contents of 2.60%, 2.51%, and 2.40% for Rh‐TiO₂/CNF, TiO₂/CNF, and CNF, respectively (Table S2, Supporting Information).

**Figure 3:**
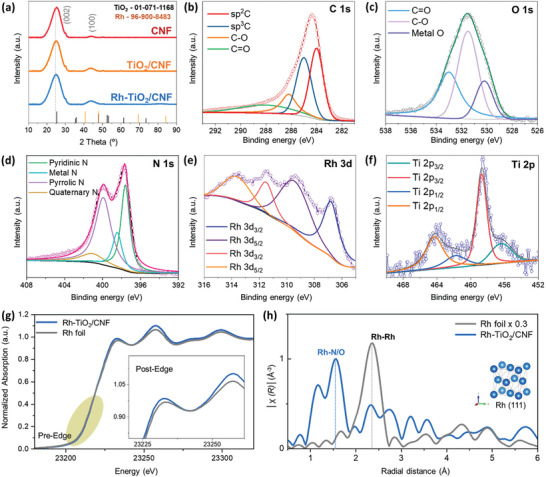
Structural characterization: a) XRD spectra of Rh‐TiO_2_/CNF, TiO_2_/CNF, and CNF. High‐resolution XPS deconvoluted spectra of b) C1s, c) O1s, d) N1s, e) Rh 3d, and f) Ti 2p of Rh‐TiO_2_/CNF. g) XANES and h) FT‐EXAFS spectra of Rh foil and Rh‐TiO_2_/CNF.

X‐ray diffraction (XRD) spectra in **Figure** **3**a illustrate the structural analyses of Rh‐TiO_2_/CNF, TiO_2_/CNF, and CNF. Broader peaks appeared ≈26.5 ° and 42.1 °, corresponding to the (002) and (100) planes of graphitic carbon derived from the CNF.^[^ ^38^ ^]^ Due to the significantly low metal loading, identifying the Rh and TiO_2_ metallic positions was challenging. Therefore, additional Raman analysis was performed to verify the structural characterization for TiO_2_ phase. Raman spectra of Rh‐TiO_2_/CNF and TiO_2_/CNF show peaks at 144, 394, 514, and 636 cm^−1^, corresponding to the E_g1_, B_1g_, A_1g_, and E_g3_ vibrations of anatase TiO_2_ phases (Figure S6, Supporting Information).^[^ ^39^ ^]^ The relative abundance of the (001) facet of anatase TiO_2_ is significantly augmented by the symmetric E_g_ and A_1g_ antisymmetric vibration of O‐Ti‐O and Ti‐O‐Ti.^[^ ^40^ ^]^ Thus, TiO_2_ is present in the anatase phase in both Rh‐TiO_2_/CNF and TiO_2_/CNF. The intensity ratio (I_D_/I_G_) of the D (≈1340 cm^−1^) and G (≈1590 cm^−1^) bands from the deconvoluted Raman spectra in Figure S7 (Supporting Information) shows values of 1.15, 1.05, and 0.94 for Rh‐TiO_2_/CNF, TiO_2_/CNF, and CNF, respectively. In Rh‐TiO_2_/CNF, the formation of the SA phase leads to a more defective structure, whereas TiO_2_/CNF and CNF exhibit similar defect structures. Additional detailed chemical composition, valence state, and structural information were explored using XPS analysis. Figure S8 (Supporting Information) presents the X‐ray photoelectron spectroscopy (XPS) survey spectra of Rh‐TiO_2_/CNF, TiO_2_/CNF, and CNF, detailing the presence of Rh, Ti, O, and C elements. For Rh‐TiO_2_/CNF, Figure 3b shows the carbon C 1s deconvolution spectra, revealing the binding energies at 284.5 and 285.3 eV, attributed to sp^2^ carbon and sp^3^ carbon, respectively. Oxidized carbon lattices of C‐O and C = O were identified ≈286.4 and 289.5 eV due to the effect of TiO_2_ and surface oxidation traces.^[^ ^41^ ^]^ The oxygen O 1s spectra in Figure 3c show C = O and C ‐O derivative O appearing ≈533.5 and 531.9 eV, respectively. The peak ≈530.5 eV indicates oxygen functionalization on the metallic Ti and Rh.^[^ ^42^ ^]^ The XPS spectrum of N 1s was deconvoluted, revealing four distinct peaks at 397.6, 398.8, 399.7, and 401.2 eV. These peaks correspond to pyridinic N, metal N, pyrrolic N, and quaternary N spectra, as shown in Figure 3d.^[^ ^43^, ^44^, ^45^ ^]^ Notably, Rh‐TiO_2_/CNF demonstrated a higher atomic ratio of pyridinic N (39.5%), suggesting that these pyridinic N sites are crucial for coordinating with the SA phase. Additionally, the presence of a peak at a binding energy of 398.8 eV, corresponding to N atoms coordinated by metal, confirms the existence of Rh─N bonds within the CNF. The Rh 3d spectra in Figure 3e were deconvoluted into two primary components. The peaks corresponding to metallic Rh^0^ associated with Rh nanoparticles appear at 306.8 and 311.6 eV for Rh 3d_3/2_. Concurrently, the formation of Rh^3+^ in the Rh SA through interaction with N was observed ≈309.1 and 313.6 eV for Rh 3d_5/2_.^[^ ^34^ ^]^ The Ti 2p spectra were deconvoluted into two major peaks of Ti 2p_3/2_ and Ti 2p_1/2._ The intense peaks at ≈458.6 and 456.4 eV correspond to Ti^4+^ valence states, while the weaker peaks ≈456.3 and 462.1 are attributed to Ti^3+^ valence states (Figure 3f).^[^ ^46^ ^]^ Detailed XPS studies of the comparative materials TiO_2_/CNF and CNF can be found in Figures S9, S10 (Supporting Information).

To further elucidate the structure of the Rh SA configuration, X‐ray absorption near‐edge structure (XANES) and extended X‐ray absorption fine structure (EXAFS) analyses were performed on Rh‐TiO_2_/CNF using synchrotron radiation. As shown in Figure 3g, the Rh *K*‐edge XANES spectra reveal that the absorption edge for Rh‐TiO_2_/CNF is observed at higher energy levels compared to the reference Rh foil. Additionally, the higher energy shift of Rh‐TiO_2_/CNF suggests a more positive valence state compared to Rh foil, indicating the presence of Rh^3+^, which is consistent with the Rh 3d XPS results.^[^ ^47^ ^]^ Fourier transforms (FT) EXAFS analysis depicted in Figure 3h further reveals the radial distance (R‐space position) of Rh‐TiO_2_/CNF. Rh‐TiO_2_/CNF exhibits two major peaks at 1.57 and 2.29 Å, corresponding to Rh‐N/O and Rh‐Rh coordination, respectively. Additional comparative *K*‐space and *Q*‐space responses of Rh‐TiO_2_/CNF and Rh foil indicate significant oscillation in Rh‐TiO_2_/CNF ≈2.1–4.2 Å^−1^, further supporting the electronic modulation effects of N with metallic Rh (Figure S11, Supporting Information).

### 2.2 HER Activity Under Alkaline Condition

**Figure 4:**
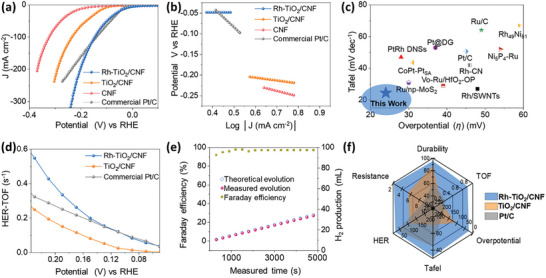
Electrocatalytic HER evaluations in conventional method: a) LSV curves and b) Tafel plots of Rh‐TiO_2_/CNF, TiO_2_/CNF, Pt/C, and CNF. c) Comparison chart of Rh‐TiO_2_/CNF with recently reported SA‐based HER in 1.0 M KOH electrolyte solution. d) Relationship between calculated TOF and applied potentials, e) Faradaic efficiency of H_2_ production. f) Overall electrochemical performance comparison web chart.

The electrochemical HER was performed in a three‐electrode cell setup. Detailed information on the electrode fabrication techniques and the configuration of the electrochemical workstation can be found in the **Experimental section** and **Supporting Information**. **Figure** **4**a shows the 75% of *IR*‐corrected steady‐state linear sweep voltammetry (LSV) polarization curves in 1.0 m KOH electrolyte solution. Remarkably, Rh‐TiO_2_/CNF exhibited a significantly low overpotential (*η*) of 24 mV at 10 mA cm^−^^2^, compared to TiO_2_/CNF (109 mV), CNF (190 mV), and even commercial Pt/C with 20 wt.% Pt (37 mV). This indicates the exceptional intrinsic activity attributed to the synergy between Rh‐N_x_ and Rh‐O_x_ on the TiO_2_/CNF substrate. The respective Tafel slopes were calculated as 24.5, 98.1, 131.6, and 38.7 mV dec^−1^ for Rh‐TiO_2_/CNF, TiO_2_/CNF, CNF, and commercial Pt/C, respectively (Figure 4b). These values indicate that Rh‐TiO_2_/CNF predominantly follows the faster Volmer‐Tafel reaction mechanism, as detailed in the Supporting Information. To further support that superior HER performance is achieved only when both Rh‐N_x_ and Rh‐O_x_ are present simultaneously, we synthesized a catalyst with only Rh‐N_x_ single atoms deposited (Rh/CNF), as shown in Figure S12 (Supporting Information). Rh/CNF was synthesized by excluding the Ti precursor during the electrospinning process. Further details are provided in the **Experimental section**. The LSV graph of Rh/CNF reveals a notable improvement in performance compared to TiO_2_/CNF; however, it still exhibits lower HER activity than Rh‐TiO_2_/CNF. Additionally, the Tafel slope of 69.8 mV indicates relatively slower kinetics. Therefore, it can be concluded that stable and efficient HER performance is only realized when both Rh‐N_x_ and Rh‐O_x_ are simultaneously present. Moreover, comparative studies of recent noble metal‐based HER activities versus our system demonstrated its practical potential for large‐scale H_2_ production (Figure 4c; Tables S7, S8, Supporting Information).

Electrochemical impedance spectroscopy (EIS) analyses further elucidated the synergistic electrode‐electrolyte interface geometry of Rh‐TiO_2_/CNF, revealing a CT resistance of 2.54 Ω, which is significantly lower than that of comparative materials by 7‐fold; 17.90 Ω for commercial Pt/C (Figure S13, Supporting Information). This indicates superior kinetics and enhanced electrocatalytic surfaces, as shown in in situ operando EIS spectra across various potential ranges, where Rh‐TiO_2_/CNF exhibits the smallest semicircle diameters at increasing potentials.^[^ ^48^ ^]^ The intrinsic activity was quantified through the turnover frequency (TOF) of H_2_ evaluations at various potentials. Rh‐TiO_2_/CNF composite exhibits a dominant catalytic activity of 0.43 s^−1^ TOF at 0.20 V versus RHE, substantially higher than that of individual TiO_2_ (2 wt%) and Pt/C (20 wt%) activities (Figure 4d). The cyclic voltammetry (CV) of each electrode is presented in Figure S14 (Supporting Information). Durability tests were conducted at high current densities of 100 and 200 mA cm^−2^ (Figure S15, Supporting Information). For a practical demonstration of H_2_ production, we employed a time‐dependent water displacement technique to quantify H_2_. The Hoffman experiment, depicted in Figure 4e, utilized Rh‐TiO_2_/CNF as the cathode and commercial IrO_2_ as the anode. The detailed method for measuring Faradaic efficiency is provided in Supporting Information. Lastly, the overall performance in a three‐electrode HER setup is shown in Figure 4f, where Rh‐TiO_2_/CNF maintained its leading performance, firmly establishing its superiority among the tested materials.

### 2.3 Theoretical Study on HER Activity

**Figure 5:**
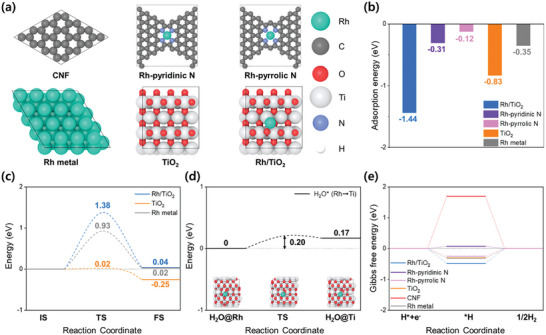
DFT calculations on HER activity: a) Geometry‐optimized atomic structures for each model. b) Water adsorption energy for each system. c) Water dissociation barrier on each surface. d) Water diffusion barrier energy from the Rh site to the Ti site in the Rh/TiO_2_ model for HER. e) Gibbs free energy diagram for HER on each system.

To investigate the effect of Rh introduction, the HER mechanism at each active site was analyzed through DFT calculations. The models consist of bare CNF, Rh‐pyridinic N, and Rh‐pyrrolic N, which are Rh SAs coordinated to pyridine‐type N and pyrrole‐type N, respectively. Additionally, Rh metal, representing the surface of Rh nanoparticle, rutile TiO_2_, and TiO_2_ with Rh SA were analyzed (**Figure** **5**a). In alkaline HER in AEMWE, water dissociation, a critical step in the Volmer mechanism, plays a key role in determining HER performance. Therefore, water adsorption behavior, which initiates the Volmer step, was investigated. Water does not adsorb on bare CNF (indicated by a positive adsorption energy) but adsorbs on Rh‐pyridinic N and Rh‐pyrrolic N, with adsorption energies of −0.31 and −0.12 eV, respectively. Strong water adsorption was also observed on Rh metal, TiO_2_, and Rh/TiO_2_, with adsorption energies of −0.35, −0.83, and −1.44 eV, respectively (Figure 5b). Thus, in addition to Rh SAs, the presence of Rh nanoparticles and TiO_2_ surfaces appears to be beneficial for enhancing water adsorption. The water dissociation barrier energy, another key factor in the Volmer step, was calculated using the climbing image nudged elastic band (CI‐NEB) method. Since neither CNF nor Rh SA in CNF provides adjacent sites for H^*^ + OH^*^ adsorption, only TiO_2_, Rh/TiO_2_, and Rh metal were considered. The nudged elastic band (NEB) image profiles for the dissociation of a water molecule into H^*^ + OH^*^ at adjacent sites are shown in Figure S16 (Supporting Information). The calculated barrier energies for water dissociation on Rh metal, Rh/TiO_2_, and TiO_2_ were 0.92, 1.38, and 0.02 eV, respectively, indicating that water dissociation is nearly barrier‐free on TiO_2_ (Figure 5c).

Rh introduction plays a crucial role in promoting water adsorption on the Rh/TiO_2_ surface. Once adsorbed, water diffuses from the Rh site to the Ti site on TiO_2_ with a low diffusion barrier of 0.20 eV (Figure 5d). This ensures efficient water transport and facilitates dissociation on TiO_2_, enhancing HER performance under alkaline conditions. The hydrogen adsorption behavior, another important aspect of HER, was also studied. The catalytic performance for HER was analyzed by calculating the Gibbs free energy for hydrogen adsorption (Figure 5e). Hydrogen adsorption models for each system are shown in Figure S17 (Supporting Information). The Gibbs free energies of hydrogen adsorption for bare CNF and TiO_2_ were 1.70 and −0.30 eV, respectively. With Rh introduction, the hydrogen adsorption energies were −0.31, 0.08 eV, −0.24, and −0.48 eV for Rh metal, Rh‐pyridinic N, Rh‐pyrrolic N, and Rh/TiO_2_, respectively. Notably, Rh‐pyridinic N, with a Gibbs free energy close to the ideal value of 0 eV, is believed to be the source of high HER performance. Consequently, the DFT calculations clearly reveal that Rh incorporation enhances HER activity through multiple mechanisms. In addition to the multiple reactive Rh sites, the TiO_2_ surfaces contribute favorably to water adsorption and facilitate near barrier‐less dissociation. Furthermore, Rh coordinated with pyridinic N exhibits optimal hydrogen adsorption, significantly enhancing its effectiveness for hydrogen evolution.

### 2.4 AEMWE Performance

**Figure 6:**
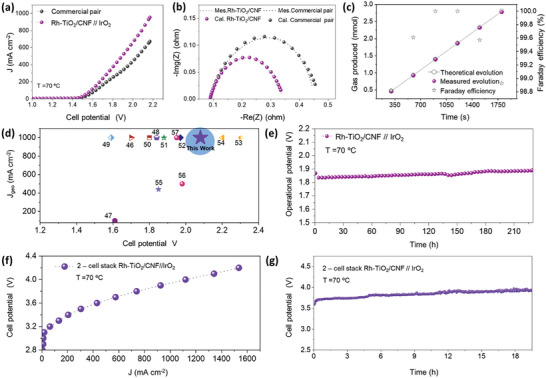
AEMWE full cell evaluations: a) LSV curves, b) EIS spectra of Rh‐TiO_2_/CNF//IrO_2_, and the commercial electrode pair at 70 °C, c) real‐time Faradic efficiency of H_2_ production, d) comparison bar chart of Rh‐TiO_2_/CNF AEMWE performance with recent reports, e) long‐term AST of Rh‐TiO_2_/CNF//IrO_2_ at 70 °C, f) *I–V* curve of the AEMWE stack cell, and g) long‐term AST of the 2‐cell stack with Rh‐TiO_2_/CNF//IrO_2._

In the full cell evaluation to eliminate *iR*‐drop in the cables, the voltage output was directly measured at the endplates. Figure S18a,b (Supporting Information) demonstrates 5 cm^2^ active area electrodes loaded single‐cell (1‐cell) AEMWE configuration. **Figure** **6**a shows the obtained *I–V* curves for the Rh‐TiO_2_/CNF//IrO_2_ and commercial Pt/C// IrO_2_ pairs of single‐cell AEMWE performance, measured at 1.0 mV s^−1^ scan rate. The catalyst loading for the cathode (Rh‐TiO_2_/CNF and commercial Pt/C) is 3.5 mg cm^−2^ while that for the anode (IrO_2_) is 2.5 mg cm^−2^. The single‐cell using Rh‐TiO_2_/CNF exhibited a significantly higher current density compared to the commercial Pt/C with 20 wt.% Pt (953 vs 672 mA cm^−2^ at 2.2 V_cell_). Specifically, the Rh‐TiO_2_/CNF electrode delivered an increase of 281 mA cm^−2^ in current density at the same potential of 2.2 V_cell_ compared to the cell employing the commercial Pt/C. EIS of single‐cell was conducted in the frequency range of 100 kHz –10 mHz with an AC voltage amplitude of 10 mV, and the data were fitted using Z‐fit (Bio‐logic) software (Figure 6b; Figure S18c, Supporting Information). To confirm that the current generated was solely from the HER, we calculated the faradaic efficiency at a constant potential of 1.58 V for 30 min at a temperature of 60 °C. We measured the amount of H_2_ produced during the electrolysis process using the water‐gas displacement method and compared it to the theoretically expected amount of H_2_ evolution (Figure 6c). For the better comparison, we included a bar chart of recently reported AEMWE performance (Figure 6d; Table S9) demonstrating that our prepared Rh‐TiO_2_/CNF is a commercially viable and cost‐effective candidate for electrochemical HER.^[^ ^49^, ^50^, ^51^, ^52^, ^53^, ^54^, ^55^, ^56^, ^57^, ^58^, ^59^, ^60^ ^]^ Figure 6e shows the single‐cell AEMWE long‐term stability of the Rh‐TiO_2_/CNF//IrO_2_ electrode pair assessed at a constant current density of 200 mA cm^−2^ at 60 °C for 225 h. The cell using Rh‐TiO_2_/CNF demonstrated relatively stable performance over 225 h, with a cell voltage change of only 0.028 V_cell_ after this period. These results confirm that the HER electrocatalyst, with partial oxidation, enhances the durability of the AEMWE single‐cell system. Furthermore, the applicability of the AEMWE stack system was validated by stacking and operating two single cells (2‐cell) comprising the electrode. Figure S18d–f (Supporting Information) shows a schematic illustration of Rh‐TiO_2_/CNF//IrO_2_‐based AEMWE stack cell design. The *I–V* curves of the 2‐cell stack using Rh‐TiO_2_/CNF//IrO_2_, shown in Figure 6f, demonstrate a peak areal current density of 1.5 A cm^−^^2^ at 4.2 V. Additionally, the real‐time durability of 2‐cell stack with Rh‐TiO_2_/CNF//IrO_2_ is illustrated in Figure 6g, where the system exhibited stable performance over 19.5 h. Although Rh is a costly precious metal, our system achieves high performance with a low loading of 2 wt.%, offering greater economic viability compared to commercial Pt/C catalysts (20 wt.% Pt). Additionally, the one‐pot synthesis of binary SA catalysts reduces both processing steps and costs, highlighting the scalability and commercial potential of this approach. This study highlights the real‐time feasibility of low weight percentage noble metal‐based electrocatalysts for the large‐scale commercialization of hydrogen energy.

## 3 Conclusion

The atomically dispersed binary Rh SA on TiO_2_/CNF was meticulously designed to serve as an effective electrode for AEMWEs. This innovative configuration, featuring Rh‐N_x_ and Rh‐O_x_ SAs, offers highly efficient sites for H and H_2_O, thereby delivering superior HER performance. As a result, the Rh‐TiO_2_/CNF demonstrates notable intrinsic properties, with an *η* of 24.1 mV. DFT analyses further clarify the inherent hydrogen affinity, with the detailed calculation of hydrogen and water adsorption confirming the feasibility of HER. Moreover, the catalyst achieved an outstanding areal current density of 1 A cm^−2^ and exhibited excellent durability, sustaining performance over 225 h in a single‐cell AEMWE setup. The successful application of a 2‐cell AEMWE stack under industrial‐scale conditions further validates the potential of Rh‐TiO_2_/CNF as a catalyst for large‐scale hydrogen production. This study presents an innovative preparation method for utilizing substantial loadings of noble metals to fabricate highly active materials for hydrogen production.

## Conflict of Interest

The authors declare no conflict of interest.

## Supporting information



Supporting Information

## Data Availability

The data that support the findings of this study are available from the corresponding author upon reasonable request.
