# Supplementary material for: Synergistic Configuration of Binary Rhodium Single Atoms in Carbon Nanofibers for High‐Performance Alkaline Water Electrolyzer
Source: Adv Sci (Weinh). 2024 Nov 24;12(3):2413176. doi: 10.1002/advs.202413176 (PMC11744564; doi:10.1002/advs.202413176)
Supplement: Supplementary file 1 — Supporting Information [file ADVS-12-2413176-s001.docx]

Supporting Information

**Synergistic Configuration of** **Binary Rhodium Single Atoms in Carbon Nanofibers for High-Performance Alkaline Water Electrolyzer**

*Natarajan Logeshwaran*†, *Gyuchan Kim*†, *Pandiarajan Thangavel*†, *Sun Seo Jeon, Kaliannan Thiyagarajan, Kampara Roopa Kishore, Hyunjoo Lee, Inseok Seo, Hongseok Yun, Sungho Lee*, Byung-Hyun Kim*, Young Jun Lee**

^†^These authors contributed equally to this work.

Dr. N. Logeshwaran, Dr. S. Lee, Dr. Y. J. Lee

Carbon Composite Materials Research Center

Korea Institute of Science and Technology (KIST)

92 Chudong-ro, Bongdong-eup, Wanju-gun, Jeonbuk, 55324, Republic of Korea

G. Kim, Prof. B.-H. Kim

Department of Applied Chemistry, Center for Bionano Intelligence Education and Research

Hanyang University ERICA

55 Hanyangdaehak-ro, Sangnok-gu, Ansan-si, Gyeonggi-do 15588, Republic of Korea

Dr. P.Thangavel

Department of Chemistry

Ulsan National Institute of Science and Technology (UNIST)

UNIST-gil, Eonyang-eup, Ulju-gun, Ulsan, 44919, Republic of Korea.

S. S. Jeon, Prof. H. Lee

Department of Chemical and Biomolecular Engineering,

Korea Advanced Institute of Science and Technology (KAIST)

Daejeon 34141, Republic of Korea

Dr. K. Thiyagarajan

Department of Mechanical Engineering

Ulsan National Institute of Science and Technology (UNIST)

UNIST-gil, Eonyang-eup, Ulju-gun, Ulsan, 44919, Republic of Korea

Dr. K. R. Kishore, Prof. Inseok Seo

School of Advanced Materials Engineering

Jeonbuk National University

Baekje-daero 567, Jeonju 54896, Republic of Korea.

Prof. H. Yun

Department of Chemistry

Hanyang University

222, Wangsimni-ro, Seongdong-gu, Seoul, Republic of Korea.

E-mail: sunghol@kist.re.kr

E-mail: bhkim00@hanyang.ac.kr

E-mail: youngjunlee@kist.re.kr

Experimental Section

**Chemicals**

Rhodium (II) sulfate (Rh_2_(SO_4_)_3_, 99.99 % metal basis), potassium hydroxides (KOH, 99.99 %), hydrochloric acid (HCl, ~37.4 %), sulfuric acid (H_2_SO_4_, ~98 %), titanium chloride (TiCl_4_, 99.995% trace metals basis), polyacrylonitrile (PAN) with an average molecular weight of 150,000 g/mol, polyvinylidene fluoride (PVDF) binder, *N*-methylpyrrolidone (NMP), and dimethylformamide (DMF) were purchased from Sigma-Aldrich. Ethanol and acetone were purchased from Samchun Chemicals. All chemicals were used without an additional cleaning process. All solvents and electrolytes were prepared using ultrapure Milli-Q water (18.2 MΩ cm at 25 ˚C).

**Synthesis of Rh-TiO_2_/CNF, Rh/CNF, TiO_2_/CNF, and CNF**

Carbon support was synthesized through electrospinning followed high-temperature carbonization process. 10 wt% of PAN, 2 wt% of Rh_2_(SO_4_)_3_, and 2 wt% of TiCl_4_ were sequentially dissolved in DMF solution. The mixture underwent stirring for 12 h in a water bath maintained at 60°C until a clear solution was obtained. The prepared viscous brown precursor solution was loaded into an electrospinning syringe and the nanofibers were spun for 6 h on a grounded static collector. An electrical potential of 8 kV was applied to the needle tip while maintaining a flow rate of 10 µL min^-1^, and the distance between the needle tip and the static collector was fixed at 18 cm. The as-spun nanofibers underwent a stabilization process at 280 °C temperature, involving a 2-step process. Initially, the nanofibers were maintained at 150 °C (ramp rate of 2 °C min^-1^) for 2 h, followed by holding at 280 °C (ramp rate of 2 °C min^-1^) for another 2 h. Subsequently, the samples underwent a procedure of cooling to room temperature before being pyrolyzed in Ar atmosphere. After a 30 min exposure to Ar gas, the pyrolysis process was initiated. Initially, the stabilized PAN nanofibers were heated to 350 °C (ramp rate: 2 °C min^-1^) for 30 minutes, followed by pyrolyzed at 900 °C (ramp rate: 5 °C min^-1^) for 2 h. The obtained nanofibers were cooled to ambient temperature, yielding Rh-TiO_2_/CNF for electrode preparation. To facilitate an extensive evaluation of electrochemical performance, additional samples including TiO_2_/CNF and bare CNF were prepared through the same synthetic process without the Rh and Ti-Rh sources, respectively.

**Cell electrode fabrication**

We purchased nickel foam substrate from Goodfellow as a current collector with nature of 0.9 mm thickness, 0.62 g cm^-1^ bulk density, 93 % porosity with 20 pores cm^-1^, and 99.5 % purity. Nickel foams were cut to the required size for use. They were then soaked in a solution of 75 mL water and 25 mL HCl and stirred for 15 min. Then, the solutions were sonicated for 10 min to remove the oxide layers and washed with ethanol and water for 3 times followed by drying in vacuum for overnight. For the 3-electrode cell systems, 3.0 mg of prepared electrocatalysts and commercial Pt/C were individually weighted and mortared for 10 min. Then 0.8 mg of PVDF powder and 0.2 mg of super P carbon black were added and mortared for 10 mins, afterwards 500 µL of NMP solution were added and sonicated for 2 h in ambient environment. The homogeneous catalysts slurries were coated on vacuumed substrate and dried in vacuum for 12 h.

**Reversible hydrogen electrode (RHE) calibration**

Reference electrode calibration is carried in three cell using Pt mesh as both working and counterpart and Ag/AgCl as reference electrode part in the 1.0 M KOH system. In which KOH medium is pretreated with hydrogen gas. Afterwards LSV cycles were evaluated and then concorded null current values is taken as RHE for Ag/AgCl, all the calculated potentials were equaled to RHE by using following equation.

$$E_{\left( RHE \right)}= \left( E_{\left( {Ag}/{AgCl} \right)}+0.098 V+0.059 pH \right) (1)$$

**Ohmic and capacitance resistance iR compensation:**

Applied potentials on CV, LSV and etc., were corrected with applied series resistance, calculated via respective Nyquist plots [1].

$$E_{iR corr}=E_{measured}-E_{i*series resitance} (2)$$

**HER kinetics**

Generally, the electrochemical HER process is defined as a redox reaction of electrolyte and active electrode interfaces. Based on the electrolyte pH condition, the H_2_ formation is either H_2_O dissociation or proton H^+^ reduction with a series of reaction kinetics. In the acidic medium H_2_ generation is far simple and easier due to massive proton clouds available in the electrolyte.

$$H^{+}+ e^{-} \leftrightarrow H^{*} (3)$$

$H^{*}+ H^{+}+ e^{-} \leftrightarrow H_{2} ($4)

$${2H}^{*} \leftrightarrow H_{2} (5)$$

$${2H}^{+}+ {2e}^{-} \leftrightarrow H_{2} (6)$$

Initially proton adsorbed on the electrocatalysts surface to form the H* adsorption denoted as discharge or Volmer step (Equation 3). Combining (H*) with proton and e^-^  to form a H_2_ in equation 4 known as desorption step or Heyrovsky step. Preferably hydrogen formation with two H* combination on electrocatalyst surface through Tafel step is represented in equation 5. Equation 6 shows the overall HER in acidic medium [2].

Compare to the acidic media HER process, alkaline medium hydrogen evolution is hard and complicated due to the lacking of proton availability. Alkaline HER initiate with water dissociation reaction, as per volcano plot alkaline HER process possibly changes with exchange current densities rather than ∆E_H_ (eV). It requires distinct reaction pathways compare with acetic media. It starts with H_2_O molecule dissociation to generate the protons, which involve the following series reaction steps.

$$H_{2}O+ e^{-}+ * \leftrightarrow{H_{ad}}/{H^{*}}+ {OH}^{-} (7)$$

$${H_{ad}}/{H^{*}}+ H_{2}O+ e^{-} \leftrightarrow{OH}^{-}+ H_{2}+ * (8)$$

$${2H}_{2}O+ {2e}^{-} \leftrightarrow{2OH}^{-}+H_{2} (9)$$

Generally, the oxidation or formation of adsorbed hydrogen in equation 7 is known to be Volmer step, where by simultaneous electron transfer involved equation 8 is known to Heyrovsky step and dissociative adsorption/desorption kinetics without electron transfer is known to be Tafel step [3].

**Over potential calculation**

75 % iR compensation applied LSV curves were evaluated overpotential through,

$${E vs. E}_{\left( RHE \right)}\left( \eta\right)= \left( E_{\left( RHE \right)}+E_{\left( RHE \right)}^{0}+0.059 pH \right)-1.29 V (10)$$

Where, η = over potential

**TOF calculation**

The hydrogen TOF per site of the electrocatalyst was calculated using the following,

$$\#H_{2}=\frac{\frac{Total hydrogen turnover}{{cm}^{2}geometric area}}{\frac{No.of active sites}{{cm}^{2} geometric area}} (11)$$

The total number of hydrogen turnover was calculated using formula

$$\#H_{2}=\left( j\frac{mA}{{cm}^{2}} \right)\left( \frac{\frac{1C}{s}}{1000 mA} \right)\left( \frac{1 mol e^{-}}{96485 C} \right)\left( \frac{1 mol H_{2}}{2 mol e^{-}} \right)\left( \frac{{6.023\times10}^{23}mol H_{2}}{1 mol H_{2}} \right) {3.12\times10}^{15} \frac{\frac{H_{2}}{s}}{{cm}^{2}} per \frac{mA}{{cm}^{2}} (12)$$

Further, the Ti and Rh elements content of Rh-TiO_2_/CNF electrocatalyst were quantified by using ICP-OES analysis and it was about ~ 2.4 wt% and ~ 1.8 wt%. Accordingly, the density of active sites based on the Ti and Rh is:

$$\left( \frac{2.4}{47.867}+\frac{1.8}{102.91} \right)\times\frac{1 mmol}{100 mg} \times3 \frac{mg}{{cm}^{2}} \times{6.022\times10}^{20}\frac{sites}{mmol}=1.22{\times10}^{18}{sites cm}^{-2}$$

For instance, TOF of the prepared catalyst at an overpotential of 200 mV was calculated and given below,

$$TOF=\frac{{169\times3.12\times10}^{15}\frac{\frac{H_{2}}{s}}{{cm}^{2}}}{{1.22\times10}^{18}{sites cm}^{-2}}= {0.432 s}^{-1}$$

**Faradic Efficiency**

The faradaic efficiency was calculated using Faraday's law equation reported in the literatures,

$Faradaic efficiency of {(H}_{2)})=\frac{nf*m}{Q}$ (13)

Faradic Efficiency = (nF x m)/Q

where *n* = number of electrons involved in the reaction for HER (*n* = 2)

*F* = Faraday's constant (96485.33 A s mol^−1^), *m* = moles of H_2_ produced.

**AEMWE measurement**

The anionic exchange membrane (AEM) (Sustainion® X37-50 Grade RT Membrane, Dioxide Materials) underwent pretreatment as follows. Initially, the AEM was transformed into its hydroxyl form by immersion in a 1.0 M KOH aqueous solution for 24 - 48 h at 25°C, followed by a subsequent 1 h rinse in deionized water. To prevent CO_2_ contamination, the pre-treated membrane was stored under humidified and CO_2_-free conditions. A commercial Ni-based AEMWE cell hardware (active area: 5 cm^2^) purchased from Dioxide Materials was utilized for the AEMWE analysis. Membrane electrode assembly (MEA) fabrication utilized the as-prepared catalyst coated on the substrates (CCS), sandwiching the Rh-based cathode, high surface area IrO_2_ on nickel fiber paper anode, and a commercial AEM membrane. For comparison, a commercial AEMWE was assembled with a high surface area IrO_2_ on nickel fiber paper anode and 20 wt% Pt/C on carbon paper prepared via spray coating. The assembled AEMWE was sealed with a torque of 4 N-m. For the preparation of catalyst ink, 20 mg of Rh-based catalyst powder was dispersed in 2.6 mL of isopropyl alcohol, along with 200 µL of deionized water and 120 µL of alkaline AEM ionomer solution. The mixture was sonicated for 30 - 60 min to achieve homogeneity. Subsequently, the catalyst ink was spray-coated onto carbon paper (Sigracet 29 BC, Fuel Cell Store) and dried at 80°C. The loading amounts of Rh-TiO_2_/CNF and commercial Pt/C on the carbon paper were determined by measuring the weight difference before and after the deposition process, resulting in an approximate loading of ~3.5 mg cm^-2^. Similar to the preparation of the cathode electrodes, the IrO_2_ anode was fabricated with pre-treated nickel fiber paper. The catalyst loading was 2.5 mg cm^-2^. Electrochemical performances were evaluated in a unique three electrode system using Metrohm autolab – PGSTAT302N potentiostat in ambient environment. Outer diameter of Ø = 6 mm graphite rod and Ag/AgCl in 3.0 M KCL purchased from Qrins Co., Ltd., (South Korea) were served as counter and reference electrodes respectively. During the electrochemical measurements, the temperature of the electrolyzer and electrolyte was maintained between 25 and 70 °C. Prior to analysis, the electrolyzers were electrochemically stabilized at a current density of 200 mA cm^−2^. All the electrochemical performance was measured in 1.0 M KOH electrolyte solution, and prepared electrodes were evaluated using CV at various potentials, LSV at 1 mV s^-1^, and EIS from the range of 100 kHz to 0.01 Hz with constant 10 mV amplitude. Further we additionally varied potential ranges, which was validated using Metrohm autolab EIS electrical circuit fitting operations. Chronopotentiometry continuous assessment (CCA) tests were obtained in 100 and 200 mA cm^-2^.

**Characterization**

The surface morphology of electrocatalysts were investigated by X-ray diffraction (XRD) diffractograms, collected using a Rigaku Smart Lab with a Cu Kα source. Chemical states were investigated using a K-Alpha XPS system (Thermo Fisher Scientific, USA). Scanning electron microscopy (SEM, Hitachi SU5000) and high-resolution transmission electron microscopy (TEM, Titan G2 Cube 60–300) was employed to verify the morphology. The elemental mapping images were obtained by energy-dispersive X-ray spectroscopy (EDS) in high-angle annular dark-field scanning transmission electron microscopy (HAADF-STEM) mode. Metal loading was measured by inductively coupled plasma-optical emission spectrometry (ICP-OES, Thermo Fisher Scientific iCAP 7000 series). X-ray absorption near-edge spectroscopy (XANES) and extended X-ray absorption fine structure (EXAFS) was performed in the Pohang synchrotron (PLS 8C Nano XAFS, and 10C Wide XAFS beamline).

**Computational methods**

Density functional theory (DFT) calculations were carried out using the Vienna Ab Initio Simulation Package (VASP 5.4.4).[4, 5, 6, 7] The electron exchange-correlation interactions were treated using the Perdew-Burke-Ernzerhof (PBE) functional within the generalized gradient approximation (GGA).[8] The interactions between core and valence electrons were described using projector augmented wave (PAW) pseudopotentials [9, 10]. The Kohn-Sham wavefunctions were expanded in a plane-wave basis set with a kinetic energy cutoff of 500 eV. Convergence criteria for total energy and maximum force were set to 10^−6^ eV and 0.02 eV/Å, respectively. A vacuum layer of 15 Å was introduced to prevent interactions between periodic images of the slab.

To account for the strong electron correlations in localized Ti 3d orbitals, the DFT+U approach was applied, with a correction Hubbard parameter U = 4.2 eV.[11, 12, 13, 14] Activation barrier energies were computed using the climbing image nudged elastic band (CI-NEB) method.[15]

The adsorption energy (E_ads_) was calculated using the following equation:

$E_{\mathrm{ads}} = E_{\mathrm{total}}-(E_{\mathrm{slab}} + E_{\mathrm{adsorbate}})$ (1)

where E_total_ is the total energy of the adsorption system, E_slab_ is the energy of the bare slab, and E_adsorbate_ is the energy of the adsorbed species.

The Gibbs free energy of hydrogen adsorption (ΔG_H*_) was determined using the equation:

${\Delta G}_{H*} = {\Delta E}_{H*} + {\Delta E}_{\mathrm{ZPE}} - T\Delta S$ (2)

where ΔE_H*_ is the calculated hydrogen adsorption energy, ΔE_ZPE_ is the change in zero-point energy, and TΔS accounts for the change in vibrational entropy.


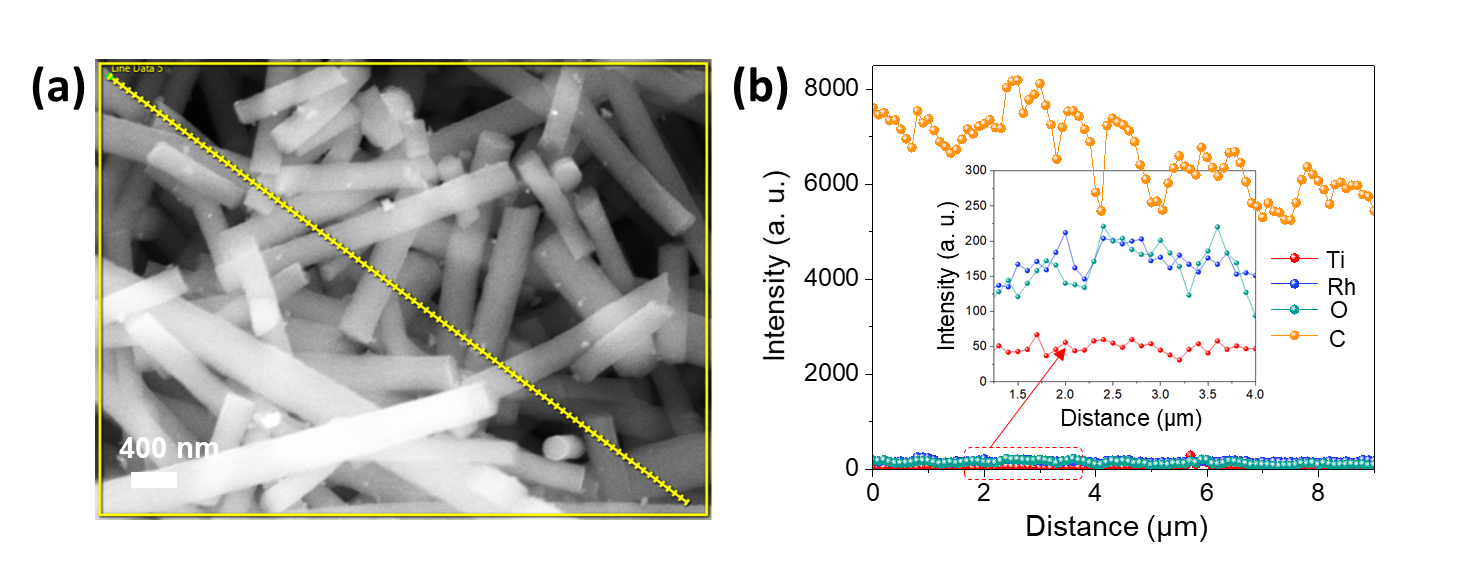


**Figure S1**. (a) SEM image and (b) selected area line scan spectrum of Rh-TiO_2_/CNF.


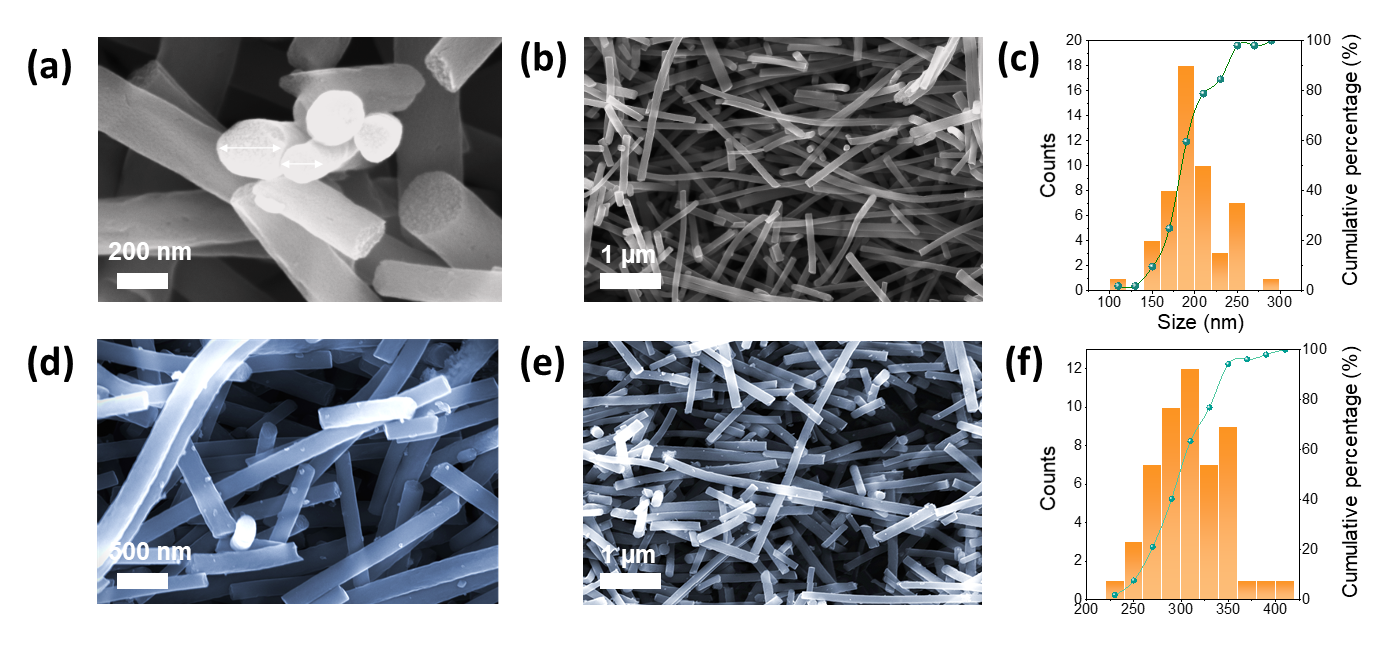


**Figure S2.** (a, b) SEM images and (c) respective average diameter population histogram of CNF, (d, e) SEM images and (f) respective average diameter population histogram of TiO_2_/CNF.


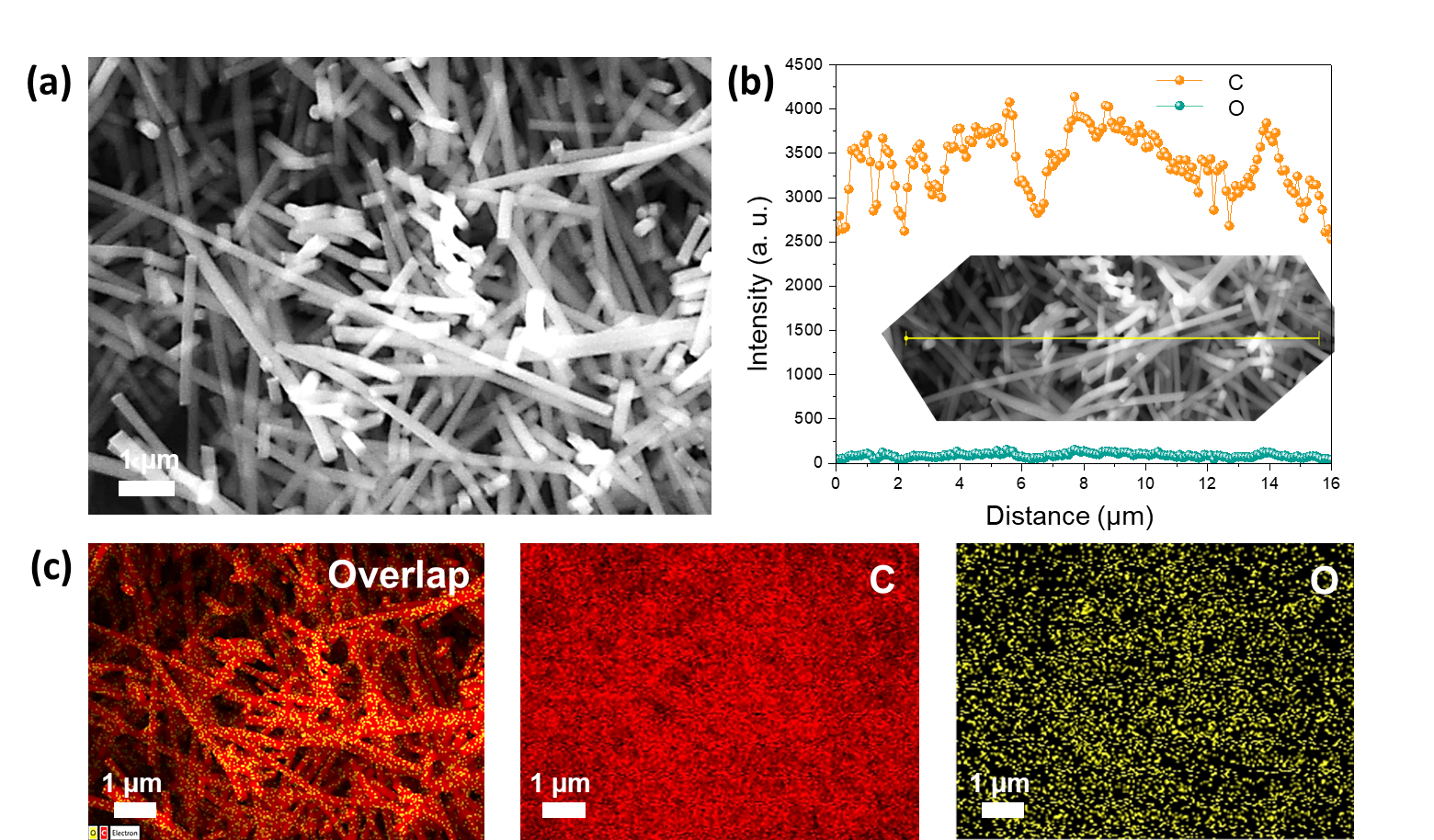


**Figure S3.** (a) SEM, (b) selected area line scan spectrum, and (c) respective elemental scan results for selected area overlap, C and O elements of CNF.


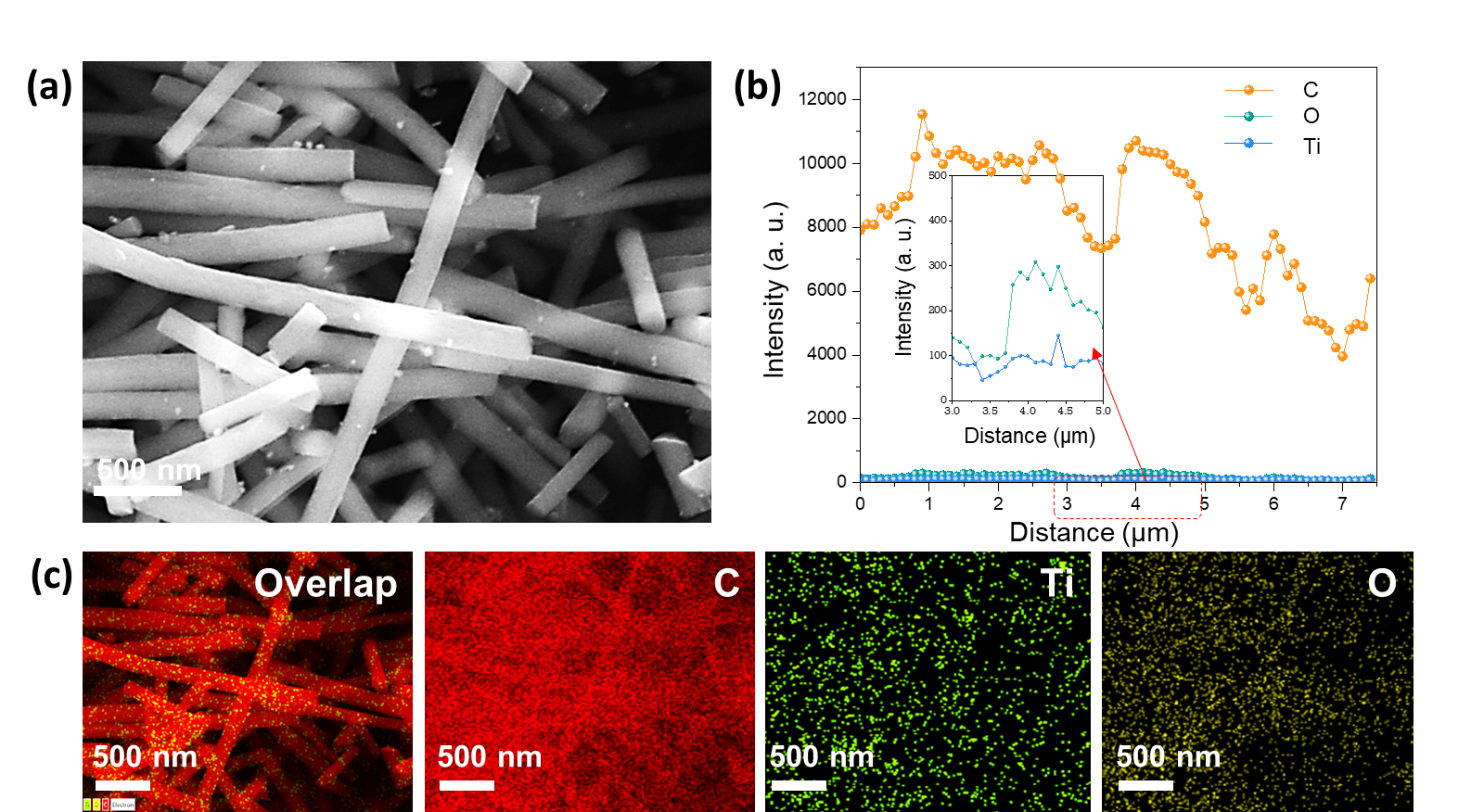


**Figure S4.** (a) SEM, (b) selected area line scan spectrum, and (c) respective elemental scan results for selected area overlap, C, Ti and O elements of TiO_2_/CNF.


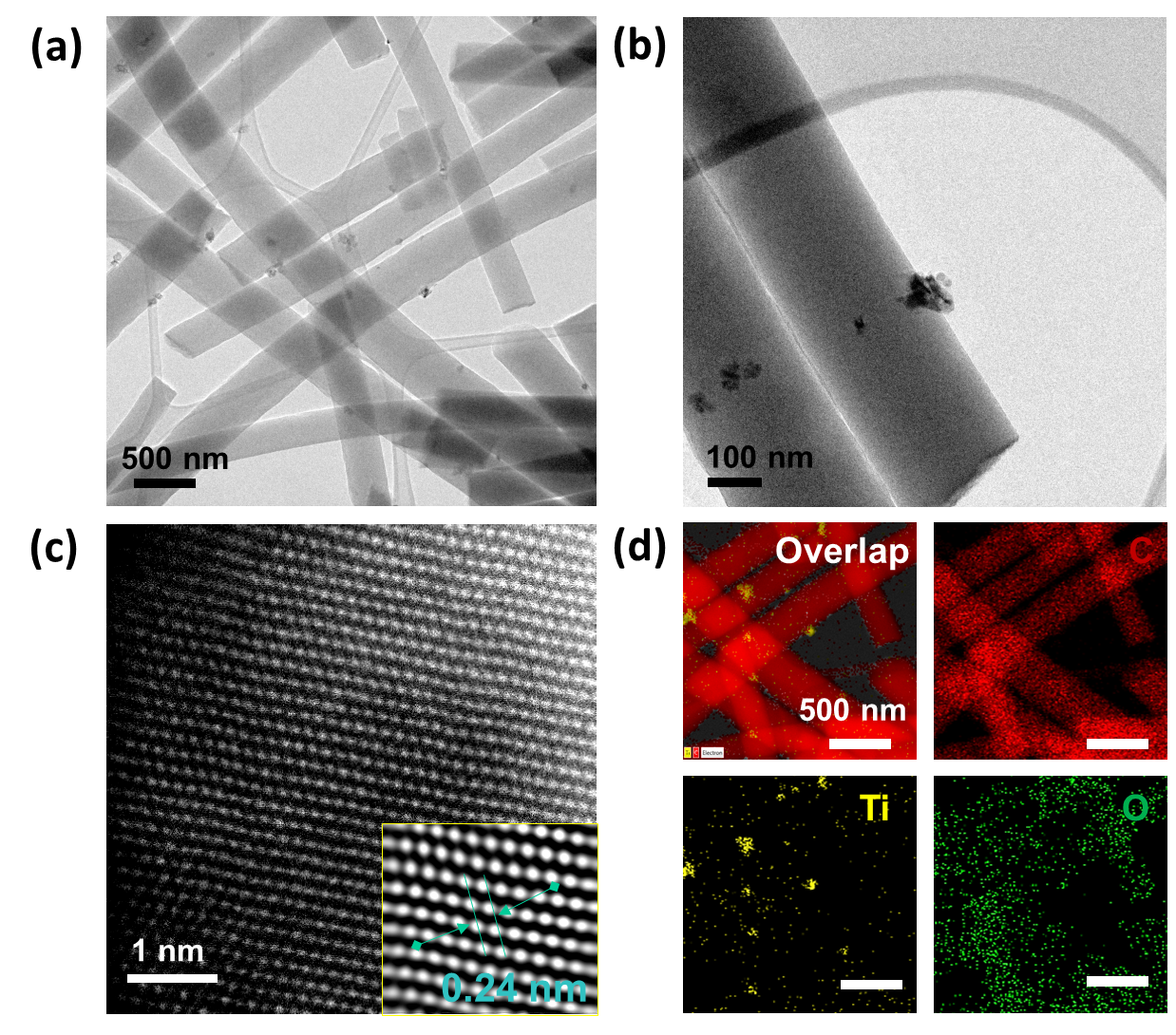


**Figure S5.** (a-b) TEM, (c) STEM atomic scale pattern images of TiO_2_/CNF (Insert images – respective inverse Fourier fast transform (IFFT) pattern for TiO_2_), and (d) EDS mapping of C, Ti, and O elements.

**Table S1.** Weight percentage of Rh-TiO_2_/CNF and TiO_2_/CNF electrocatalyst were evaluated from ICP-OES results.

|  | ICP-OES | |
| --- | --- | --- |
| **Elements** | Rh (wt%) | Ti (wt%) |
| **Rh-TiO_2_/CNF** | 1.8 | 2.4 |
| **TiO_2_/CNF** | - | 1.9 |

**Table S2.** Chemical composition of Rh-TiO_2_/CNF, TiO_2_/CNF, and CNF

|  | **N (wt %)** | **C (wt %)** | **O (wt %)** |
| --- | --- | --- | --- |
| **Rh-TiO_2_/CNF** | 2.60 | 80.80 | 3.32 |
| **TiO_2_/CNF** | 2.51 | 79.60 | 3.43 |
| **CNF** | 2.40 | 78.90 | 2.58 |

**Table S3.** XRD analysis operational conditions.

| **XRD operation conditions** | | | |
| --- | --- | --- | --- |
| **K-Alpha_1_ wavelength** | 1.540598 | Divergence slit | 0.19 mm |
| **K-Alpha_2_ wavelength** | 1.544426 | Phi | 57.7 |
| **Ratio K-Alpha_2_/K-Alpha_1_** | 0.5 | Time per step | 85.09 |
| **Generator voltage** | 40 | Scan range | 5.006000001 – 89.50000 |
| **Tube current** | 30 | Scan step size | 0.0334225 |

**
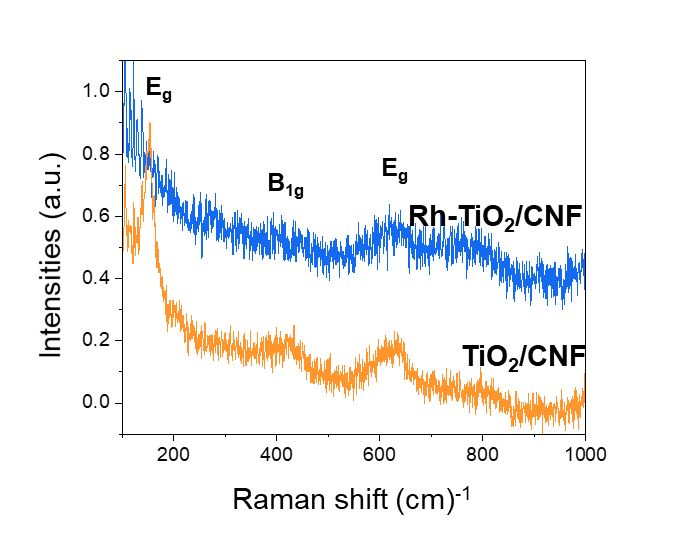
**

**Figure S6**. Raman spectra of Rh-TiO_2_/CNF and TiO_2_/CNF.


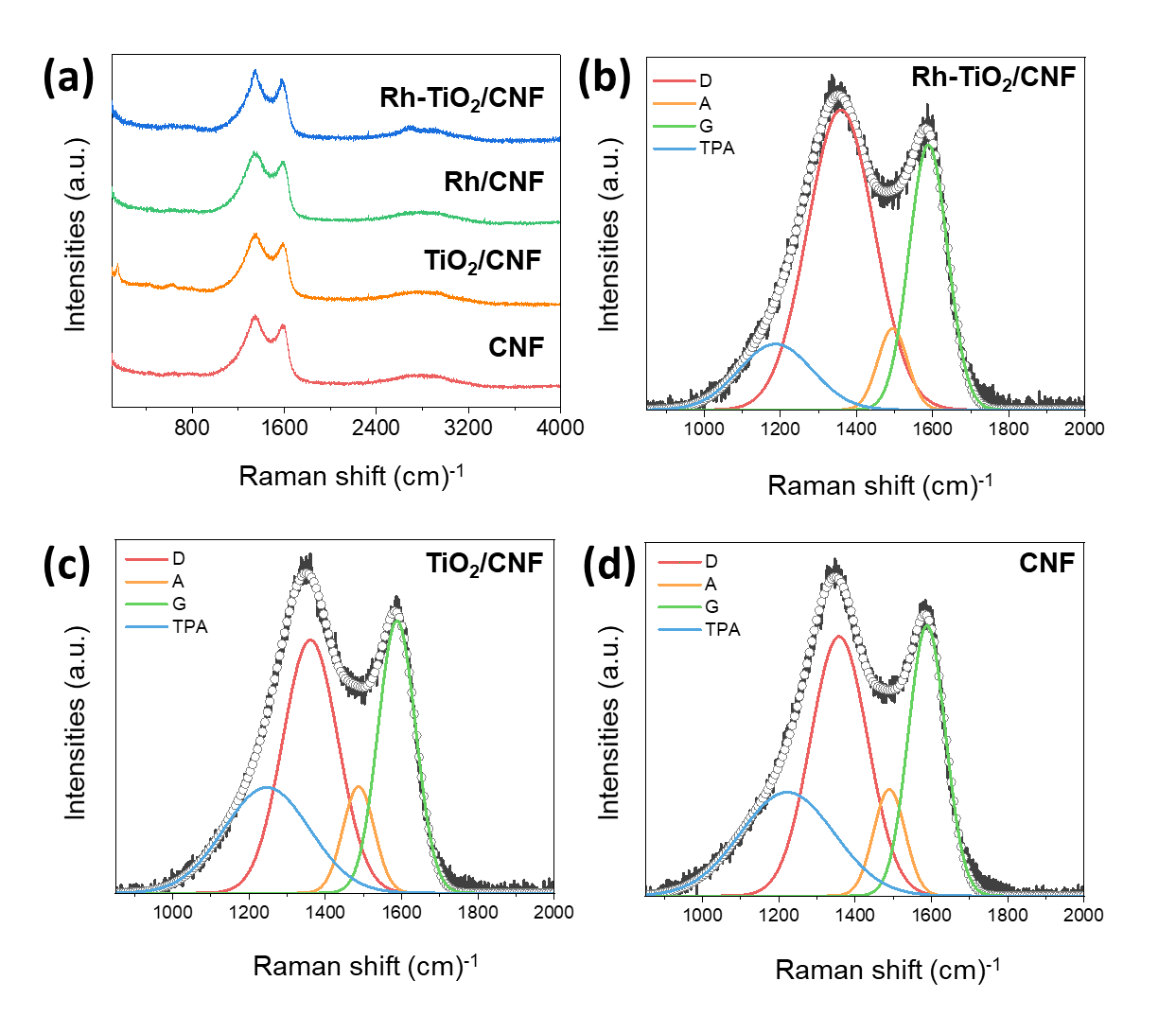


**Figure S7**. (a) Comparative Raman spectra of Rh-TiO_2_/CNF, TiO_2_/CNF, and CNF. (b-d) Raman deconvolution spectra: (b) Rh-TiO_2_/CNF, (c) TiO_2_/CNF, and (d) CNF. The deconvolution of the Raman spectra was fitted into five bands: TPA (around 1180 cm^-1^) is associated with transpolyacetylene structure. D (around 1340 cm^-1^) corresponds to the in-plane defect of graphene layers in carbon. A (around 1500 cm^-1^) and G (around 1590 cm^-1^) are related to amorphous carbon and ordered graphitic structures, respectively.


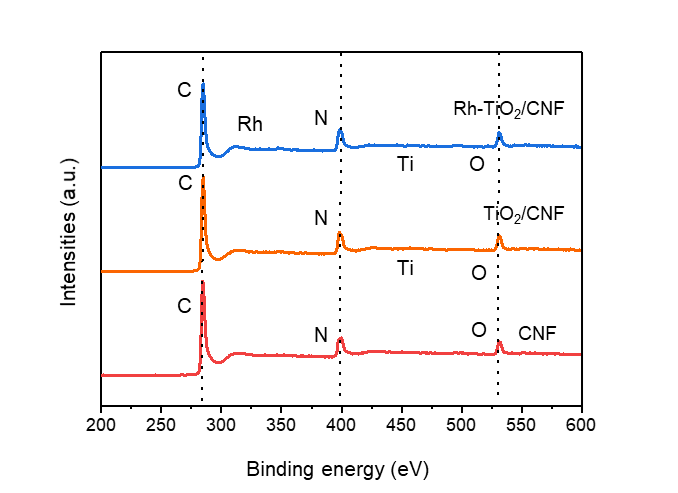


**Figure S8.** Comparative XPS survey spectrum of Rh-TiO_2_/CNF, TiO_2_/CNF, and CNF.


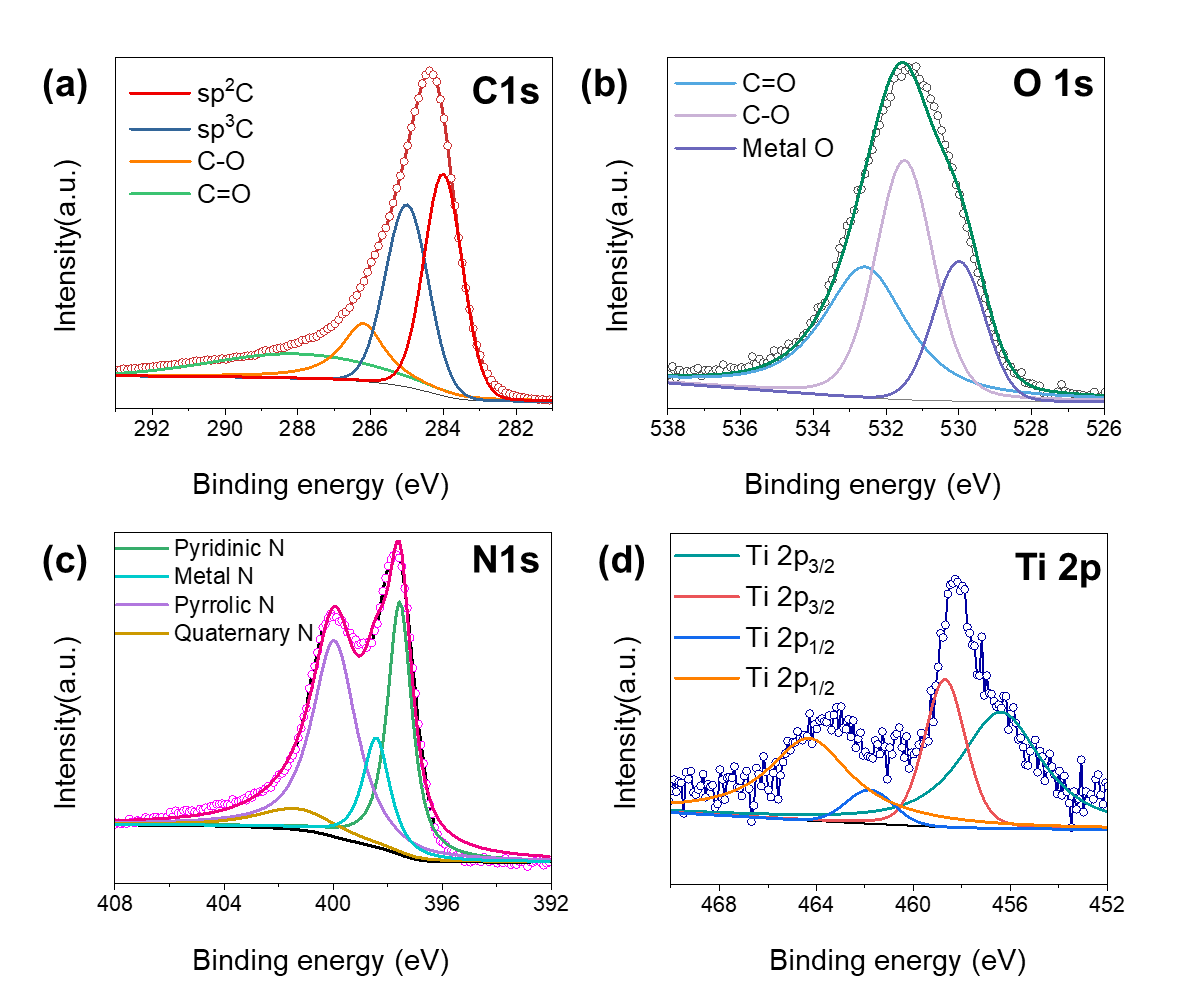


**Figure S9**. High-resolution XPS deconvolution of TiO_2_/CNF: (a) C 1s, (b) O 1s, (c) N 1s, and (d) Ti 2p.


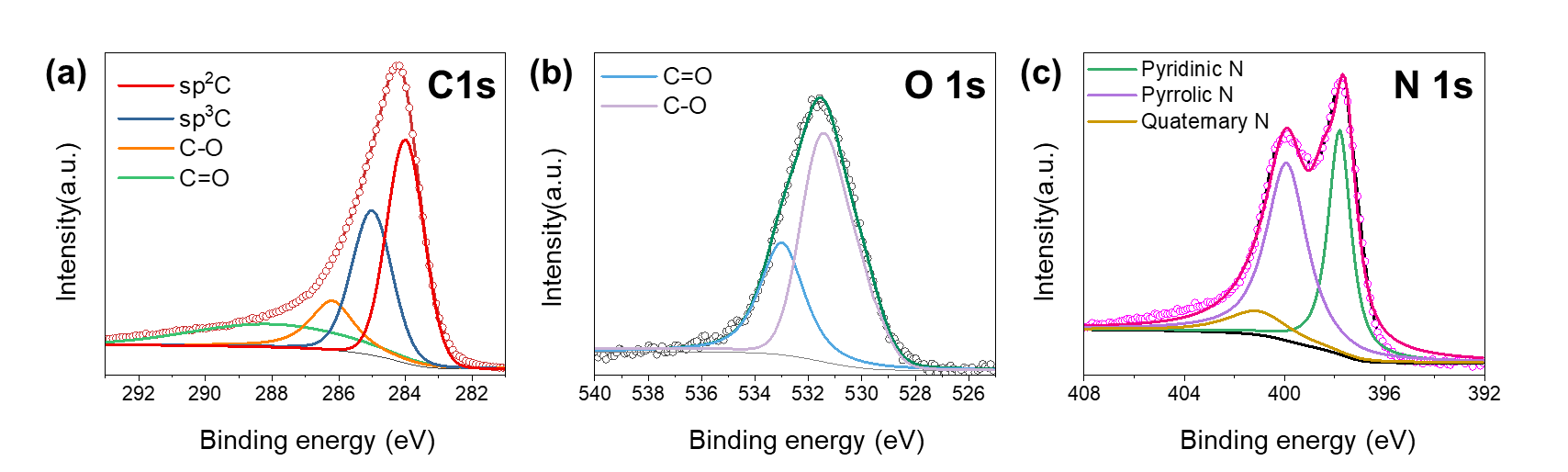


**Figure S10**. High-resolution XPS deconvolution of CNF: (a) C 1s, (b) O 1s, and (c) N 1s.

**Table S4.** Elemental composition of Rh-TiO_2_/CNF evaluated by XPS.

| **Name** | **Peak BE  (eV)** | **FWHM  (eV)** | **Area  (P)** | **Atomic  (%)** |
| --- | --- | --- | --- | --- |
| **C 1s** | 284.41 | 2.06 | 318577.5 | 77.11 |
| **Rh 3d** | 309.4 | 7.54 | 19228.14 | 1.28 |
| **N 1s** | 398.12 | 2.36 | 103836.6 | 15.31 |
| **Ti 2p** | 458.49 | 1.53 | 6043.91 | 0.96 |
| **O 1s** | 531.46 | 3.24 | 51887.21 | 5.14 |

**Table S5.** Elemental composition of TiO_2_/CNF evaluated by XPS.

| **Name** | **Peak BE  (eV)** | **FWHM  (eV)** | **Area  (P)** | **Atomic  (%)** |
| --- | --- | --- | --- | --- |
| **C 1s** | 284.4 | 1.97 | 350459.55 | 79.01 |
| **N 1s** | 398.07 | 2.41 | 105676.69 | 14.34 |
| **Ti 2p** | 458.23 | 1.94 | 7152.22 | 1.28 |
| **O 1s** | 531.32 | 3.15 | 56207.02 | 5.24 |

| **Name** | **Peak BE (eV)** | **FWHM  (eV)** | **Area  (P)** | **Atomic  (%)** |
| --- | --- | --- | --- | --- |
| **C 1s** | 284.31 | 2.05 | 360168.9 | 80.53 |
| **N 1s** | 397.97 | 2.6 | 103606.4 | 14.91 |
| **O 1s** | 531.58 | 3.18 | 49252.23 | 4.56 |

**Table S6.** Elemental composition of CNF evaluated by XPS

**
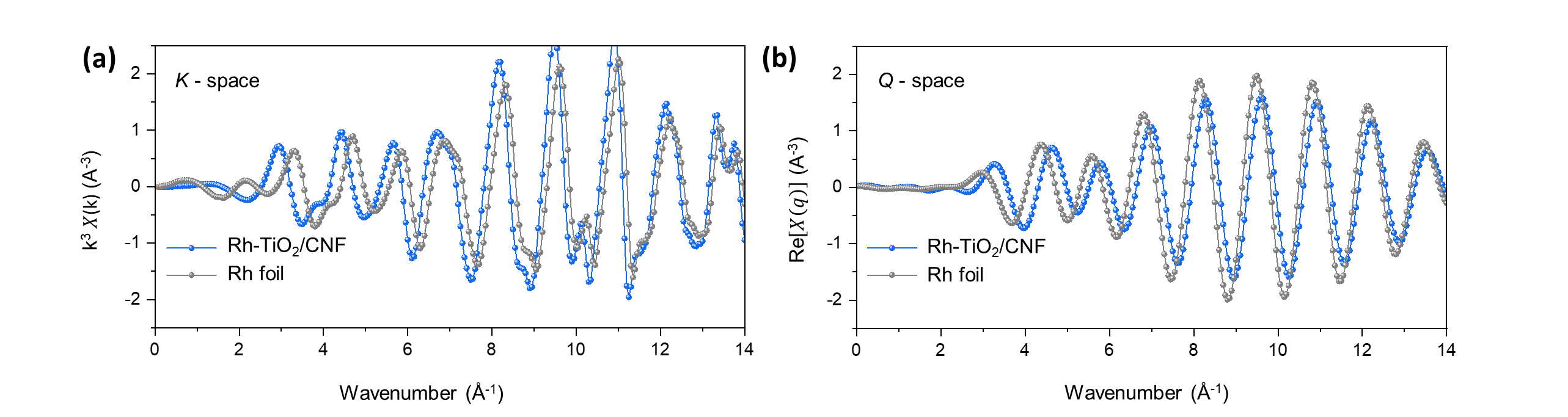
**

**Figure S11.** (a) Respective *K* space and (b) *Q* space responses of Rh *K*-edge for Rh-TiO_2_/CNF and Rh foil.


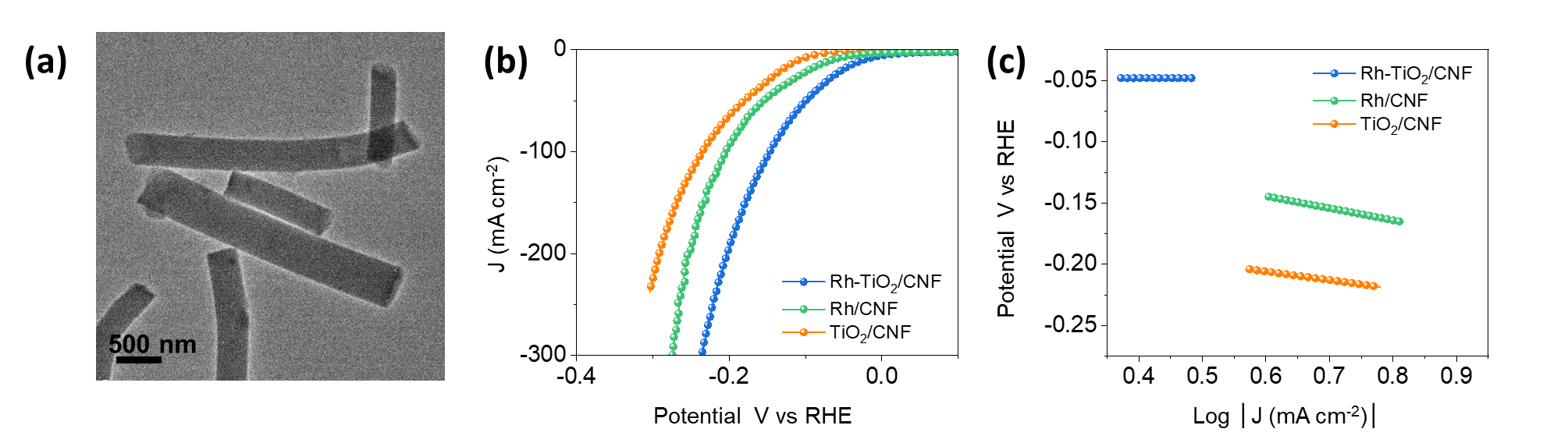


**Figure S12.** (a) TEM image of Rh/CNF. (b) LSV curve and (c) Tafel plots of Rh-TiO_2_/CNF, Rh/CNF, and TiO_2_/CNF.

**Table S7.** Comparison table of HER in conventional three electrode systems.

| **Material** | **Electrolyte** | **η overpotential**  **(mV)** | **Tafel**  **(mV dec^-1^)** | **Ref.** |
| --- | --- | --- | --- | --- |
| **Rh-TiO_2_/CNF** | **1.0 M KOH** | **24.5** | **24** | **This work** |
| 1wt% Rh-WO_3_ | Alkaline seawater | 318 | 166 | [16] |
| 5wt% Rh-WO_3_ | Alkaline seawater | 98 | 84 |  |
| Rh/C | 1.0 M KOH | 171 | 89.4 | [17] |
| Rh-CN | 1.0 M KOH | 46 | 42.0 |  |
| Cs_3_Rh_2_I_9_/NC-R | 1.0 M KOH | 25 | 30.3 | [18] |
| Rh_3_Tb IMs | 1.0 M KOH | 19 | 22.2 | [19] |
| PtRh DNAs | 1.0 M KOH | 28 | 47 | [20] |
| Rh90/CP | 0.5 M H_2_SO_4_ | 10.9 | 32 | [21] |
| Co-NCNFs-5Rh | 1.0 M KOH | 13 | 27.2 | [22] |
| Rh/SWNTs | 1.0 M KOH | 48 | 27 | [23] |
| Rh NSs | 1.0 M KOH | 63 | 107.2 | [24] |
| Rh–Rh_3_Se_4_/C | 1.0 M KOH | 29 | 110.6 | [25] |
| Rh_49_Ni_51_ | 1.0 M KOH | 59 | 67 | [26] |
| CoPt-PtSA | 1.0 M KOH | 31 | 43.65 | [27] |
| Ni5P4-Ru | 1.0 M KOH | 54 | 52 | [28] |
| Pt@DG | 1.0 M KOH | 37 | 53 | [29] |
| Ru/np-MoS2 | 1.0 M KOH | 30 | 31 | [30] |
| Vo-Ru/HfO2-OP | 1.0 M KOH | 39 | 29 | [31] |
| Commercial Pt/C | 1.0 M KOH | 45 | 50.7 | [32] |
| Commercial Ru/C | 1.0 M KOH | 49 | 64.0 |  |

**Table S8.** Comparison of HER performance in conventional three-electrode systems with recently reported SA catalysts

| **Electrocatalysts** | **Metal loading**  **(wt %)** | **Electrolyte** | **η overpotential**  **(mV)** | **Tafel**  **(mV dec^-1^)** | **Ref.** |
| --- | --- | --- | --- | --- | --- |
| **Rh-TiO_2_/CNF** | **~ 2.4** | **1.0 M KOH** | **24.5** | **24** | **This work** |
| Pt-MoS_2_ | 3 | 0.5 M H_2_SO_4_ | 67.4 | 76.2 | [33] |
| Ti_3_C_2_Tx-Pt_SA_ | 0.84 | 0.5 M H_2_SO_4_ | 38 | 45 | [34] |
| Pt-SSC | 10 | 0.5 M H_2_SO_4_ | 76 | / | [35] |
| 10 Pt/VS_2_/CP | 3.16 | 0.5 M H_2_SO_4_ | 73 | 39.46 | [36] |
| Pt_1_/NMHCS | 1.59 | 0.5 M H_2_SO_4_ | 41 | 56 | [37] |
| Pt/RuCeO_x_-PA | 0.49 | 0.5 M H_2_SO_4_ | 41 | 31 | [38] |
| Pt_SA_/N-C | 2.1 | 0.5 M H_2_SO_4_ | 130 | 67 | [39] |
| Pt-SAs/WS_2_ | 4.1 | 0.5 M H_2_SO_4_ | 32 | 28 | [40] |
| Pt_1_-Mo_2_C-C | 0.7 | 1.0 M KOH | 155 | 64 | [41] |
| Pt-V_2_CTx- | 0.88 | 1.0 M KOH | 68.1 | 98.6 | [42] |
| PtSA/MoS_2_ | 0.68 | 1.0 M KOH | 123 | 76.71 | [43] |
| SA Pt-Ti_3_C_2_ | 7.7 | 1.0 M KOH | 59 | 36 | [44] |
| W_1_Mo_1_-NG | 7.5 | 1.0 M KOH | 67 | 45 | [29] |
| Pt/C (20wt.%) | ~20 | 1.0 M KOH | 45 | 50.7 | [32] |


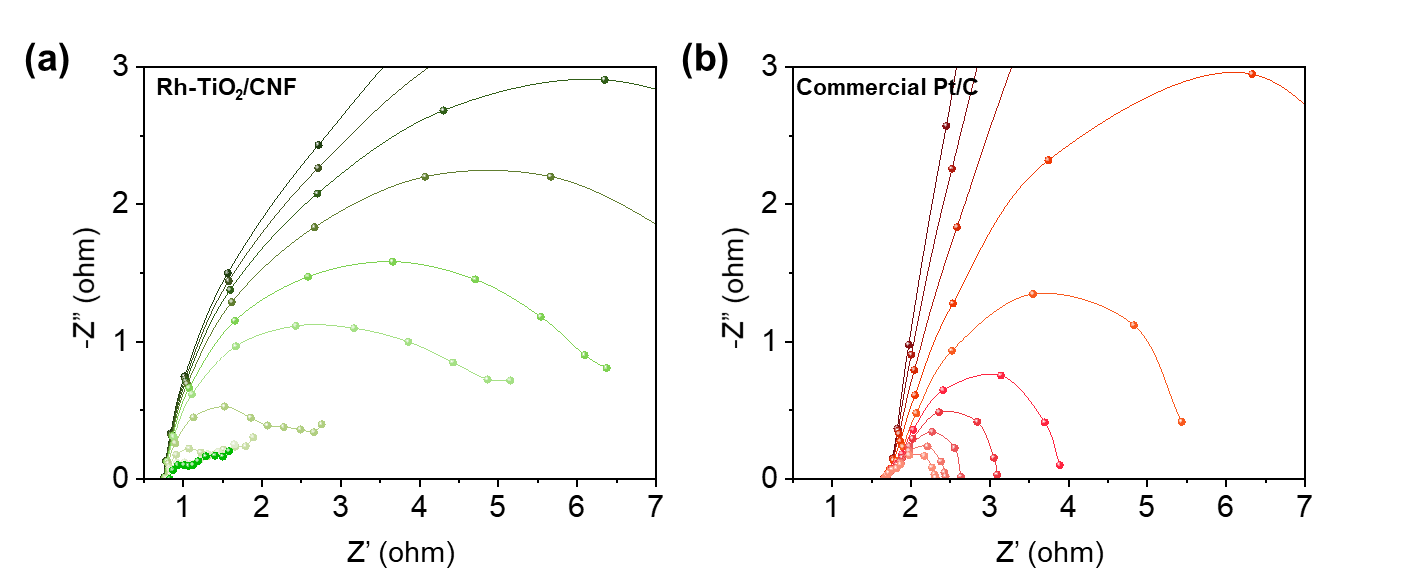


**Figure S13**. Various applied potential Nyquist plots of (a) Rh-TiO_2_/CNF and (b) commercial Pt/C in 1.0 M KOH solution.


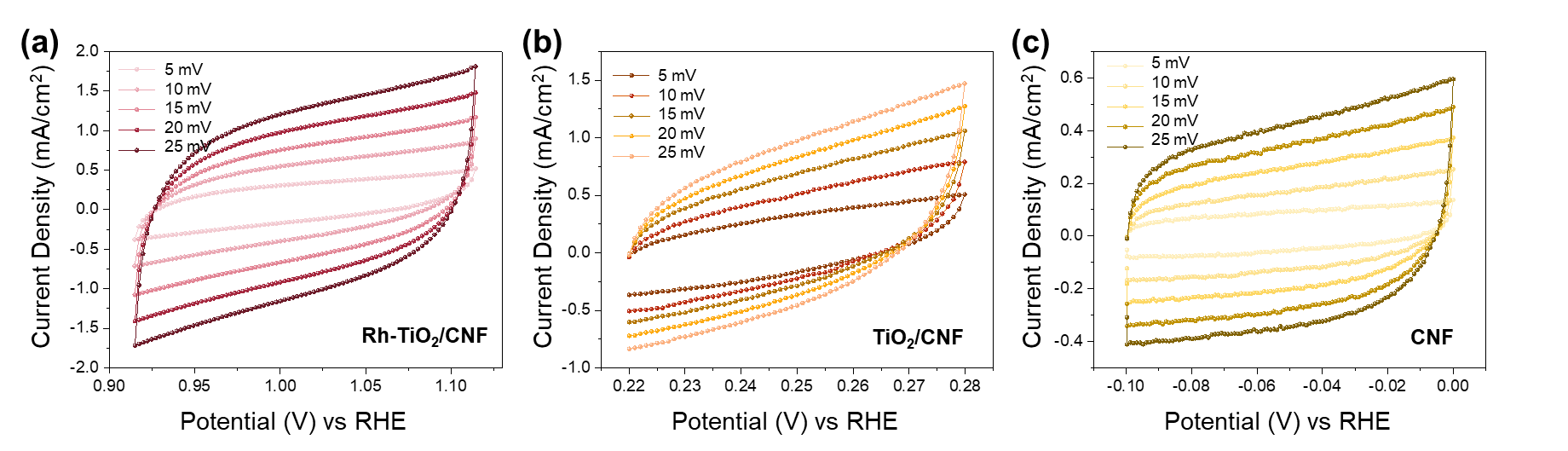


**Figure S14.** Non-faradaic region cyclic voltammetry patterns of (a) Rh-TiO_2_/CNF, (b) TiO_2_/CNF, and (c) CNF in various scan rates 5-25 mV in 1.0 M KOH solution.

**
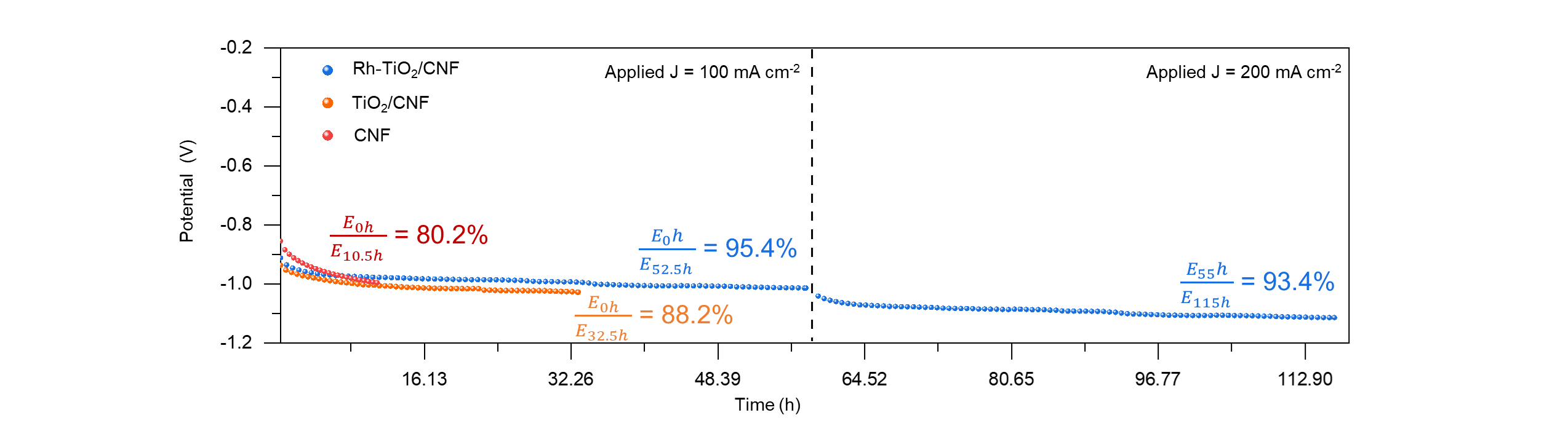
**

**Figure S15.** Long-term durable chronopotentiometry tests for Rh-TiO_2_/CNF, TiO_2_/CNF, and CNF at applied current density of 100 mA cm^-2^ and 200 mA cm^-2^ in 1.0 M KOH solution.

**
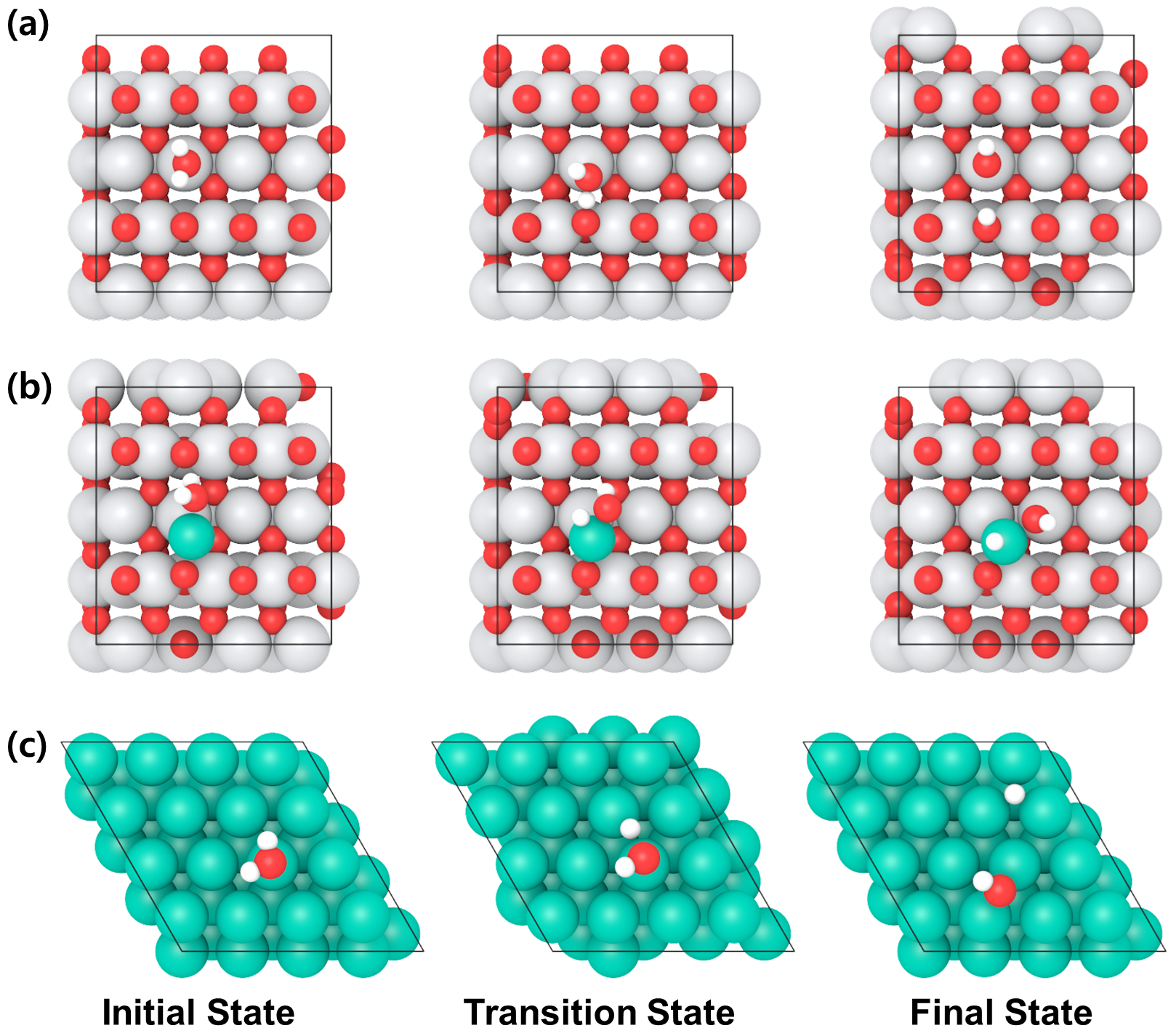
 Figure S16.** Geometry-optimized structures illustrating the water dissociation behavior on (a) TiO_2_, (b) Rh/TiO_2_, and (c) Rh metal.


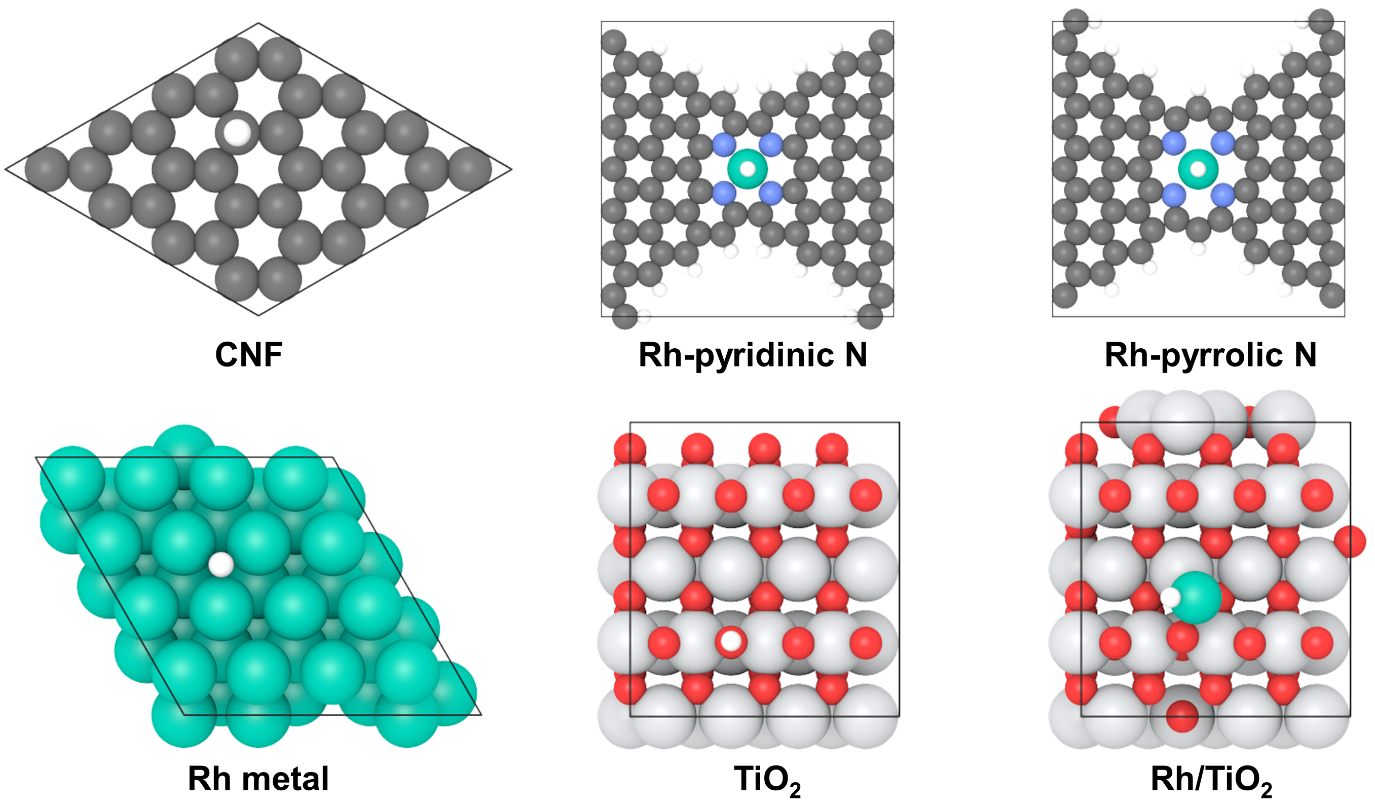


**Figure S17.** Geometry-optimized models of hydrogen adsorption on each system.


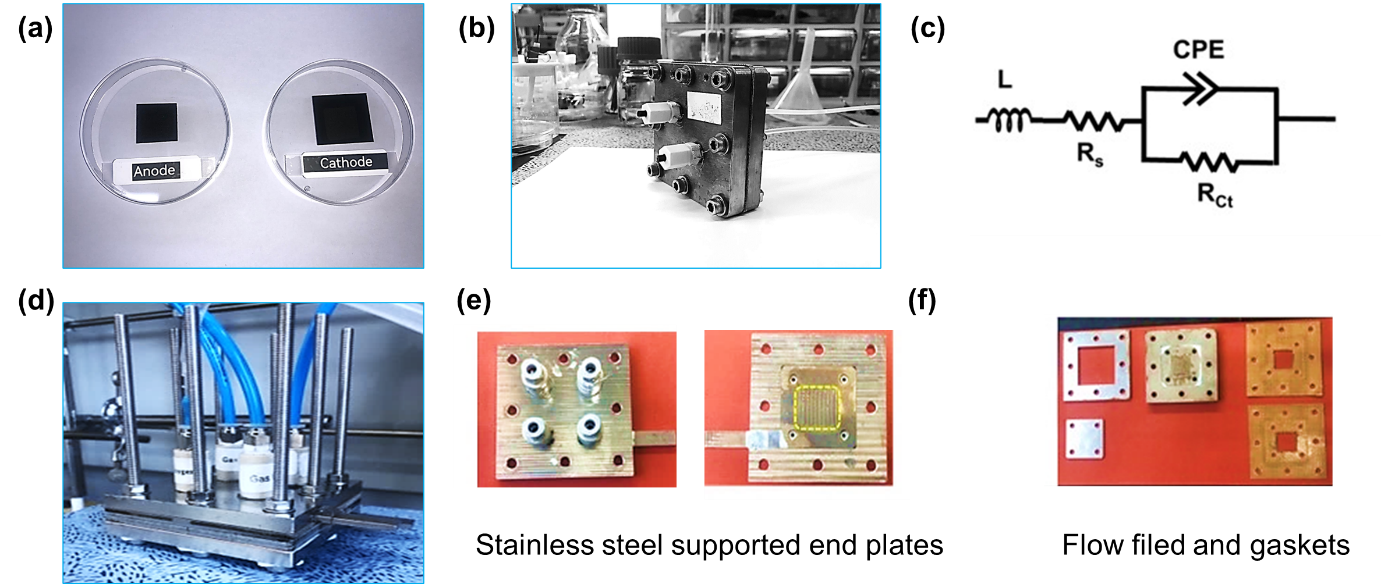


**Figure S18.** AEMWE setup: (a) Spray-coated anode with 20 wt % commercial IrO_2_ and a cathode using Rh-TiO_2_/CNF, (b) single-cell AEMWE setup with IrO_2_//Rh-TiO_2_/CNF configuration, (c) equivalent electronic circuit model applied to the single cell AEMWE performance for EIS analysis, (d) 2-cell stack AEMWE setup with IrO_2_//Rh-TiO_2_/CNF, and (e-f) Detailed internal components of the corresponding stack AEMWE setup.

**Table S9.** Comparison of AEMWE performance with recently reported SA catalysts

| **Electrocatalysts** | **Electrolyte** | **T**  **(Cº)** | **J**  **(A cm^-2^)** | **E**  **(V)** | **Ref.** |
| --- | --- | --- | --- | --- | --- |
| **Rh-TiO_2_/CNF//**  **IrO_2_** | **1.0 M KOH** | **70** | **1.0** | **2.05** | **This work** |
| Pt_1_/CoHPO//Pt_1_/CoHPO | 1.0 M KOH | 80 | 1.0 | 1.8 | [45] |
| Pt/C//RuO_2_ commercial | 1.0 M KOH | 70 | 1.0 | 2.42 | [46] |
| np/Pt_1_Ru_1_Ni_0.85_Se//np/Pt_1_Ru_1_-Ni_0.85_Se | 1.0 M KOH | 60 | 1.0 | 1.84 | [47] |
| Pt-Ru (10 wt.%) SWNT | 0.1 M KOH | 45 | 0.5 | 1.72 | [48] |
| S-FeNiOOH\|\|P-Fe_3_O_4_-x | 1.0 M KOH | 60 | 1.0 | 1.97 | [49] |
| P-Pt/NiMoO_4_@NF | 1.0 M KOH | 60 | 0.5 | 1.75 | [50] |
| AS-Ni_3_S_2_/Co_3_S_4_ | 1.0 M KOH | 60 | 1.0 | 1.71 | [51] |
| Ru/Zn-N-C | 1.0 M KOH | 60 | 1.0 | 1.83 | [52] |
| PtC_60_ | 1.0 M KOH | 60 | 1.0 | 2.01 | [53] |
| Pt NCs | 1.0 M KOH | 60 | 1.0 | 2.58 | [53] |
| Ru-LC-Ni(OH)_2_//FeNi LDH | 1.0 M KOH | 80 | 1.0 | 1.69 | [54] |
| Co-SA/CC | 3.0 M KOH | 60 | 1.0 | 2.06 | [55] |
| Co-P_0.43_@NF//IrO_2_ | 1.0 M KOH | 60 | 1.0 | 1.76 | [56] |
| 1%Pt−NiCoP@MXene//NiFe LDH | 1.0 M KOH | 50 | 1.0 | 1.95 | [57] |

**References:**

[1] P. Thangavel, G. Kim, K. S. Kim, *Journal of Materials Chemistry A* **2021**, *9* (24), 14043.

[2] G. Zhao, K. Rui, S. X. Dou, W. Sun, *Advanced Functional Materials* **2018**, *28* (43), 1803291.

[3] B. Huang, R. R. Rao, S. You, K. Hpone Myint, Y. Song, Y. Wang, W. Ding, L. Giordano, Y. Zhang, T. Wang, S. Muy, Y. Katayama, J. C. Grossman, A. P. Willard, K. Xu, Y. Jiang, Y. Shao-Horn, *JACS Au* **2021**, *1* (10), 1674.

[4] G. Kresse, J. Furthmüller, *Physical review B* **1996**, *54* (16), 11169.

[5] *Computational materials science* **1996**, *6* (1), 15.

[6] G. Kresse, J. Hafner, *Physical review B* **1993**, *47* (1), 558.

[7] *Physical Review B* **1994**, *49* (20), 14251.

[8] J. P. Perdew, K. Burke, M. Ernzerhof, *Physical review letters* **1996**, *77* (18), 3865.

[9] G. Kresse, D. Joubert, *Physical review b* **1999**, *59* (3), 1758.

[10] P. E. Blöchl, *Physical review B* **1994**, *50* (24), 17953.

[11] V. I. Anisimov, J. Zaanen, O. K. Andersen, *Physical Review B* **1991**, *44* (3), 943.

[12] V. I. Anisimov, I. Solovyev, M. Korotin, M. Czyżyk, G. Sawatzky, *Physical Review B* **1993**, *48* (23), 16929.

[13] I. Solovyev, P. Dederichs, *Physical Review B* **1994**, *49* (10), 6736.

[14] B. J. Morgan, G. W. Watson, *Surface Science* **2007**, *601* (21), 5034.

[15] G. Henkelman, B. P. Uberuaga, H. Jónsson, *The Journal of chemical physics* **2000**, *113* (22), 9901.

[16] N.-A. Nguyen, E. Chuluunbat, T. A. Nguyen, H.-S. Choi, *International Journal of Hydrogen Energy* **2023**, *48* (84), 32686.

[17] B. Jiang, A. Huang, T. Wang, Q. Shao, W. Zhu, F. Liao, Y. Cheng, M. Shao, *Journal of Colloid and Interface Science* **2020**, *571*, 30.

[18] G. Lin, Z. Zhang, Q. Ju, T. Wu, C. U. Segre, W. Chen, H. Peng, H. Zhang, Q. Liu, Z. Liu, Y. Zhang, S. Kong, Y. Mao, W. Zhao, K. Suenaga, F. Huang, J. Wang, *Nature Communications* **2023**, *14* (1), 280.

[19] Q. Li, C. Sun, H. Fu, S. Zhang, X. Sun, J.-C. Liu, Y. Du, F. Luo, *Small* **2023**, *n/a* (n/a), 2307052.

[20] Z. Han, R.-L. Zhang, J.-J. Duan, A.-J. Wang, Q.-L. Zhang, H. Huang, J.-J. Feng, *International Journal of Hydrogen Energy* **2020**, *45* (11), 6110.

[21] J. Kim, H. Kim, S. H. Ahn, *ACS Sustainable Chemistry & Engineering* **2019**, *7* (16), 14041.

[22] X. Chen, W. Li, C. Wang, X. Lu, *Journal of Colloid and Interface Science* **2023**, *650*, 304.

[23] W. Zhang, X. Zhang, L. Chen, J. Dai, Y. Ding, L. Ji, J. Zhao, M. Yan, F. Yang, C.-R. Chang, S. Guo, *ACS Catalysis* **2018**, *8* (9), 8092.

[24] N. Zhang, Q. Shao, Y. Pi, J. Guo, X. Huang, *Chemistry of Materials* **2017**, *29* (11), 5009.

[25] S. Pan, S. Ma, C. Chang, X. Long, K. Qu, Z. Yang, *Materials Today Physics* **2021**, *18*, 100401.

[26] K. Kani, H. Lim, A. E. Whitten, K. Wood, A. J. E. Yago, M. S. A. Hossain, J. Henzie, J. Na, Y. Yamauchi, *Journal of Materials Chemistry A* **2021**, *9* (5), 2754.

[27] W. Yang, P. Cheng, Z. Li, Y. Lin, M. Li, J. Zi, H. Shi, G. Li, Z. Lian, H. Li, *Advanced Functional Materials* **2022**, *32* (39), 2205920.

[28] Q. He, D. Tian, H. Jiang, D. Cao, S. Wei, D. Liu, P. Song, Y. Lin, L. Song, *Advanced Materials* **2020**, *32* (11), 1906972.

[29] Q. Yang, H. Liu, P. Yuan, Y. Jia, L. Zhuang, H. Zhang, X. Yan, G. Liu, Y. Zhao, J. Liu, S. Wei, L. Song, Q. Wu, B. Ge, L. Zhang, K. Wang, X. Wang, C.-R. Chang, X. Yao, *Journal of the American Chemical Society* **2022**, *144* (5), 2171.

[30] K. Jiang, M. Luo, Z. Liu, M. Peng, D. Chen, Y.-R. Lu, T.-S. Chan, F. M. F. de Groot, Y. Tan, *Nature Communications* **2021**, *12* (1), 1687.

[31] G. Li, H. Jang, S. Liu, Z. Li, M. G. Kim, Q. Qin, X. Liu, J. Cho, *Nature Communications* **2022**, *13* (1), 1270.

[32] Y. Zhu, M. Klingenhof, C. Gao, T. Koketsu, G. Weiser, Y. Pi, S. Liu, L. Sui, J. Hou, J. Li, H. Jiang, L. Xu, W.-H. Huang, C.-W. Pao, M. Yang, Z. Hu, P. Strasser, J. Ma, *Nature Communications* **2024**, *15* (1), 1447.

[33] A. Shan, X. Teng, Y. Zhang, P. Zhang, Y. Xu, C. Liu, H. Li, H. Ye, R. Wang, *Nano Energy* **2022**, *94*, 106913.

[34] J. Zhang, E. Wang, S. Cui, S. Yang, X. Zou, Y. Gong, *Nano Letters* **2022**, *22* (3), 1398.

[35] P. Tang, P.-Y. Huang, J. E. N. Swallow, C. Wang, D. Gianolio, H. Guo, J. H. Warner, R. S. Weatherup, M. Pasta, *ACS Catalysis* **2023**, *13* (14), 9558.

[36] J. Zhu, L. Cai, X. Yin, Z. Wang, L. Zhang, H. Ma, Y. Ke, Y. Du, S. Xi, A. T. S. Wee, Y. Chai, W. Zhang, *ACS Nano* **2020**, *14* (5), 5600.

[37] P. Kuang, Y. Wang, B. Zhu, F. Xia, C.-W. Tung, J. Wu, H. M. Chen, J. Yu, *Advanced Materials* **2021**, *33* (18), 2008599.

[38] T. Liu, W. Gao, Q. Wang, M. Dou, Z. Zhang, F. Wang, *Angewandte Chemie International Edition* **2020**, *59* (46), 20423.

[39] J. Wang, H.-Y. Tan, T.-R. Kuo, S.-C. Lin, C.-S. Hsu, Y. Zhu, Y.-C. Chu, T. L. Chen, J.-F. Lee, H. M. Chen, *Small* **2021**, *17* (16), 2005713.

[40] Y. Shi, Z.-R. Ma, Y.-Y. Xiao, Y.-C. Yin, W.-M. Huang, Z.-C. Huang, Y.-Z. Zheng, F.-Y. Mu, R. Huang, G.-Y. Shi, Y.-Y. Sun, X.-H. Xia, W. Chen, *Nature Communications* **2021**, *12* (1), 3021.

[41] S. Niu, J. Yang, H. Qi, Y. Su, Z. Wang, J. Qiu, A. Wang, T. Zhang, *Journal of Energy Chemistry* **2021**, *57*, 371.

[42] S. Park, Y.-L. Lee, Y. Yoon, S. Y. Park, S. Yim, W. Song, S. Myung, K.-S. Lee, H. Chang, S. S. Lee, K.-S. An, *Applied Catalysis B: Environmental* **2022**, *304*, 120989.

[43] J. Zhu, Y. Tu, L. Cai, H. Ma, Y. Chai, L. Zhang, W. Zhang, *Small* **2022**, *18* (4), 2104824.

[44] T. Chu, G. Wang, X. Zhang, Y. Jia, S. Dai, X. Liu, L. Zhang, X. Yang, B. Zhang, F.-Z. Xuan, *Nano Letters* **2024**, *24* (31), 9666.

[45] L. Zeng, Z. Zhao, F. Lv, Z. Xia, S.-Y. Lu, J. Li, K. Sun, K. Wang, Y. Sun, Q. Huang, Y. Chen, Q. Zhang, L. Gu, G. Lu, S. Guo, *Nature Communications* **2022**, *13* (1), 3822.

[46] Y. Zhao, M. Sun, Q. Wen, S. Wang, S. Han, L. Huang, G. Cheng, Y. Liu, L. Yu, *Journal of Materials Chemistry A* **2022**, *10* (18), 10209.

[47] L. Cai, H. Bai, C.-w. Kao, K. Jiang, H. Pan, Y.-R. Lu, Y. Tan, *Small* **2024**, *20* (26), 2311178.

[48] F. S. M. Ali, R. L. Arevalo, M. Vandichel, F. Speck, E.-L. Rautama, H. Jiang, O. Sorsa, K. Mustonen, S. Cherevko, T. Kallio, *Applied Catalysis B: Environmental* **2022**, *315*, 121541.

[49] Q. Yu, Y. Fu, Z. Liu, X. Liu, L. Guo, T. Wang, J. Chi, Z. Wu, L. Wang, *Applied Catalysis B: Environment and Energy* **2025**, *361*, 124598.

[50] S. Xu, J. Chi, T. Cui, Z. Li, F. Liu, J. Lai, L. Wang, *Nano Energy* **2024**, *126*, 109698.

[51] H. Zhang, N. Li, S. Gao, A. Chen, Q. Qian, Q. Kong, B. Y. Xia, G. Hu, *eScience* **2024**, 100311.

[52] Y. Wan, W. Chen, S. Wu, S. Gao, F. Xiong, W. Guo, L. Feng, K. Cai, L. Zheng, Y. Wang, R. Zhong, R. Zou, *Advanced Materials* **2024**, *36* (11), 2308798.

[53] J. Chen, M. Aliasgar, F. B. Zamudio, T. Zhang, Y. Zhao, X. Lian, L. Wen, H. Yang, W. Sun, S. M. Kozlov, W. Chen, L. Wang, *Nature Communications* **2023**, *14* (1), 1711.

[54] L. Wang, M. Ma, C. Zhang, H.-H. Chang, Y. Zhang, L. Li, H.-Y. Chen, S. Peng, *Angewandte Chemie International Edition* **2024**, *63* (7), e202317220.

[55] P. Zhao, C. Peng, Q. Zhang, X. Fan, H. Chen, Y. Zhu, Y. Min, *Chemical Engineering Journal* **2023**, *461*, 142037.

[56] Y. Shi, M. Wang, D. Zhang, H. Li, C. Li, T. Zhan, J. Lai, L. Wang, *Advanced Functional Materials* **2024**, *n/a* (n/a), 2410825.

[57] H.-J. Niu, C. Huang, T. Sun, Z. Fang, X. Ke, R. Zhang, N. Ran, J. Wu, J. Liu, W. Zhou, *Angewandte Chemie International Edition* **2024**, *63* (20), e202401819.
